# Quantum Quality with Classical Cost: *Ab Initio* Nonadiabatic Dynamics Simulations Using the Mapping Approach to Surface Hopping

**DOI:** 10.1021/acs.jpclett.4c00535

**Published:** 2024-05-23

**Authors:** Jonathan R. Mannouch, Aaron Kelly

**Affiliations:** Hamburg Center for Ultrafast Imaging, Universität Hamburg and the Max Planck Institute for the Structure and Dynamics of Matter, Luruper Chaussee 149, 22761 Hamburg, Germany

## Abstract

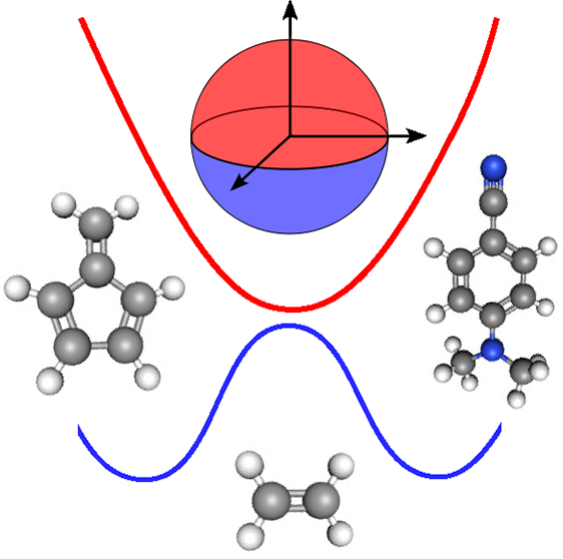

Nonadiabatic dynamics methods are an essential tool for investigating photochemical processes. In the context of employing first-principles electronic structure techniques, such simulations can be carried out in a practical manner using semiclassical trajectory-based methods or wave packet approaches. While all approaches applicable to first-principles simulations are necessarily approximate, it is commonly thought that wave packet approaches offer inherent advantages over their semiclassical counterparts in terms of accuracy and that this trait simply comes at a higher computational cost. Here we demonstrate that the mapping approach to surface hopping (MASH), a recently introduced trajectory-based nonadiabatic dynamics method, can be efficiently applied in tandem with *ab initio* electronic structure. Our results even suggest that MASH may provide more accurate results than on-the-fly wave packet techniques, all at a much lower computational cost.

Accurate computational simulations are crucial for understanding and interpreting experiments that investigate photoexcited molecular processes. Such systems are often high dimensional, involving multiple electronic potential energy surfaces and reaction channels, which makes constructing accurate parametrized models extremely challenging and time-consuming. Hence, performing *ab initio* simulations “on-the-fly”, such that the surfaces are computed in tandem with the propagation of the nuclear degrees of freedom, is often the only feasible option. In order to make such simulations practical, the number of electronic-structure calls per time step must be kept minimal, which requires dynamical approaches based on trajectories or localized nuclear basis functions.

Most dynamics approaches have not been specifically tested for *ab initio* simulations. Instead, these methods are commonly benchmarked on simplified models for which numerically exact quantum results can be generated.^1−4^ However, it is often unclear how far the conclusions from these simplified models can be extended to more realistic systems. Recently, a test set of photoexcited molecular systems have been proposed as a benchmark for investigating the properties of different nonadiabatic dynamics algorithms using on-the-fly *ab initio* electronic structure.^5^ These three molecules—ethylene, 4-(*N*,*N*-dimethylamino)benzonitrile (DMABN), and fulvene—were initially chosen because their dynamics show similarities to the three so-called Tully models,^6^ but their utility for benchmarking goes far beyond that. In particular, it is known that these systems give rise to different conical intersection topographies^5^ and pathways for approaching the intersections,^7^ therefore providing a rigorous test for any nonadiabatic dynamics method. This test set is currently gaining interest from the community, and it has already been the focus of a number of classical trajectory^5,8^ and Gaussian wavepacket^5,7^ studies. In this work, we ascertain the accuracy and utility of a novel trajectory-based dynamics technique for performing *ab initio* simulations, the mapping approach to surface hopping (MASH),^9^ by applying it to simulate this molecular photochemistry test set and comparing our results with other more established methods. In order to determine a hierarchy in the accuracy of these approaches, we also compare with numerically exact quantum dynamics applied to linear vibronic coupling (LVC) models previously parametrized for these molecules.^7^

Before discussing the relaxation dynamics of the three photoexcited molecular systems, we give a brief overview of the dynamics approaches used in this work with a particular emphasis on their similarities and differences. More information can be found in refs (9−12).

Fewest-switches surface hopping (FSSH)^6,10^ is the most popular independent-trajectory approach for simulating nonadiabatic dynamics in molecules. In FSSH, the nuclei are propagated according to (classical) Born–Oppenheimer molecular dynamics on a single surface, and nonadiabatic transitions are described by stochastic changes in the “active” surface, called “hops”. The hopping probability is related to the rate of change of the underlying electronic wave function, which itself is propagated according to the associated time-dependent Schrödinger equation. One issue with altering the active surface in this way is that it can become inconsistent with the electronic wave function, leading to the so-called overcoherence error that is known to significantly degrade the accuracy of the obtained results. To fix this, a number of decoherence corrections have been proposed,^13−21^ which sporadically reset the wave function to the current active surface. While not guaranteed,^22^ it is generally accepted that decoherence corrections lead to an improvement in the accuracy of the obtained results in the majority of cases.

Despite the substantial progress that has been made in understanding many foundational aspects of FSSH,^23,24^ a number of variants of the FSSH dynamics algorithm are nevertheless still widely used in the literature. The main aspect that differs between most FSSH algorithms lies in the way in which the nuclear velocities are rescaled at a hop. While it is generally agreed upon that rescaling along the nonadiabatic coupling vector (NACV) is the correct thing to do,^13,25−27^ many other schemes are used in practical implementations of FSSH.^27^ In particular, rescaling all degrees of freedom equally, which is often referred to as rescaling “along the velocity vector”, is probably the most commonly used.^28−30^ Additionally, an upward hop must be aborted if there is insufficient nuclear kinetic energy, often referred to as a “frustrated hop”. The nuclear velocity along the NACV is reflected at a frustrated hop in many FSSH implementations,^13^ although other suggestions have been made,^31−33^ along with those that try to avoid frustrated hops altogether.^34,35^

The mapping approach to surface hopping (MASH)^9^ is a recently proposed independent-trajectory approach that alleviates the problems of FSSH by utilizing the best features of mapping-based semiclassical trajectories^2,3,36,37^ in a surface hopping algorithm. In many aspects, the algorithm is identical to FSSH, but it contains the following key differences. In MASH, the active surface is not an additional parameter within the theory but is uniquely determined from the electronic wave function. For two-state problems, this corresponds to selecting the surface for which the electronic wave function has the largest associated probability. In addition, the stochastic nature of hops in FSSH is replaced by a fully deterministic dynamics which guarantees that the electronic wave function and the active surface remain consistent. The overcoherence problem is, therefore, resolved in MASH without the need for ad hoc decoherence corrections. For example, MASH accurately captures nonadiabatic thermal rates,^38^ whereas decoherence corrections are known to be needed for the analogous FSSH simulations.^33,39−42^

In fact, MASH gives a unique prescription for all aspects of the simulation algorithm, including the velocity rescaling and treatment of frustrated hops. In agreement with what many have suggested for FSSH,^13,25−27^ MASH determines that the velocity must be rescaled along the NACV at a hop and reflected in the case of a frustrated hop, in order for the approach to reproduce the short-time behavior of exact quantum dynamics. In particular, the exact quantum-mechanical equation of motion for the so-called “kinematic momentum”^43^ for mode *j* depends on the Born–Oppenheimer and nonadiabatic contributions to the nuclear force, which are given by the two terms on the right-hand side of the following equation
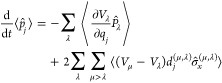
1where *V*_λ_ is the Born–Oppenheimer surface for state |ψ_λ_⟩,  is the associated electronic population operator, *d*_*j*_^(μ,λ)^ is the NACV between states |ψ_μ_⟩ and |ψ_λ_⟩ and  is the associated electronic coherence operator. More details associated with this formula are given in the Supporting Information. MASH is constructed to describe the Born–Oppenheimer (adiabatic) force through the active surface and the nonadiabatic force through the velocity rescaling performed along the NACV. In addition, MASH has already been benchmarked on a range of model systems,^38,44,45^ where it was regularly found to offer improvements over FSSH.

To summarize the above discussion, a hierarchy of the surface hopping approaches can be established according to their expected accuracy. First, it is expected that surface hopping approaches that perform the velocity rescaling along the velocity vector (FSSH-vel) will be less accurate than those that perform the velocity rescaling along the NACV (FSSH-nacv), due to the fact that the latter can describe dynamical effects arising from the nonadiabatic force. Second, fewest-switches surface hopping approaches that incorporate a decoherence correction (dFSSH) are expected to be more accurate than those that do not (FSSH), because it is important to impose consistency between the active surface and the electronic wave function. Finally, MASH is expected to be either just as accurate or more accurate than dFSSH-nacv. This hierarchy will be helpful for determining what is likely to be the correct dynamics in *ab initio* photochemical simulations, where numerically exact quantum dynamics is not obtainable.

A completely different approach for describing nonadiabatic transitions compared to the quantum-classical approaches described above is taken in *ab initio* multiple spawning (AIMS).^11,12^ Based on a series of approximations to full multiple spawning,^46,47^ AIMS is a Gaussian wavepacket algorithm that was developed for performing on-the-fly *ab initio* simulations. Gaussian basis functions are propagated classically on single Born–Oppenheimer surfaces, and new Gaussians are “spawned” whenever the system enters a region of strong nonadiabatic coupling. The evolution of the Gaussian weights is then obtained by solving an approximate time-dependent Schrödinger equation spanned by the Gaussian basis. The main advantage of AIMS over the surface hopping approaches is its coupled trajectory nature, which goes beyond the independent-trajectory approximation, albeit at an increased computational cost. On the other hand, given that AIMS only uses a minimal number of Gaussian basis functions, its accuracy will largely be determined by how effectively the Born–Oppenheimer forces are able to move the Gaussians into the correct regions of nuclear phase space. As a result, one way of improving upon AIMS is to instead propagate the Gaussians using equations of motion that ensure eq 1 is satisfied, as is done for example in the variational multiconfiguration Gaussian (vMCG) approach.^48^ Given that AIMS is not a benchmark method,^49^ the relative accuracy of AIMS compared to the quantum-classical approaches therefore depends on the relative severity of the independent-trajectory approximation for realistic molecular simulations compared to how effectively the minimal set of Gaussian basis functions generated by AIMS spans the full support of the time-dependent wave function.

We now consider in more detail the relaxation dynamics of ethylene, DMABN, and fulvene, using the same electronic structure theory for each system as defined in ref (5). The initial conditions are taken to be of the Franck–Condon type, with the electronic system on the upper of the two considered adiabats and the nuclear system in the ground vibrational state associated with the harmonic approximation to the electronic ground state potential. MOLPRO 2012^50^ and GAUSSIAN 16^51^ were used for the SA-CASSCF and LR-TDDFT electronic structure calculations, while the surface hopping and AIMS dynamics were performed using the SHARC 2.0^52−54^ and AIMS/MOLPRO^55^ codes. Files containing the electronic structure inputs and the ground-state geometries and frequencies for each system are provided in the Supporting Information. Each system displays a different type of relaxation pathway involving conical intersections. One way that this excited-state relaxation dynamics can be visualized is through the probability density of the dynamical energy gap between the two Born–Oppenheimer surfaces, as presented in the second row of Figure 1 for the MASH trajectories. In these figures, the Franck–Condon region corresponds to a large value of Δ*V*, while the conical intersection seams are about Δ*V* ≈ 0. Also given in the first row of the same figure is the effective nonadiabatic coupling between the states (i.e., the magnitude of the dot product of the NACV with the nuclear velocity) averaged over the same trajectories.

**Figure 1:**
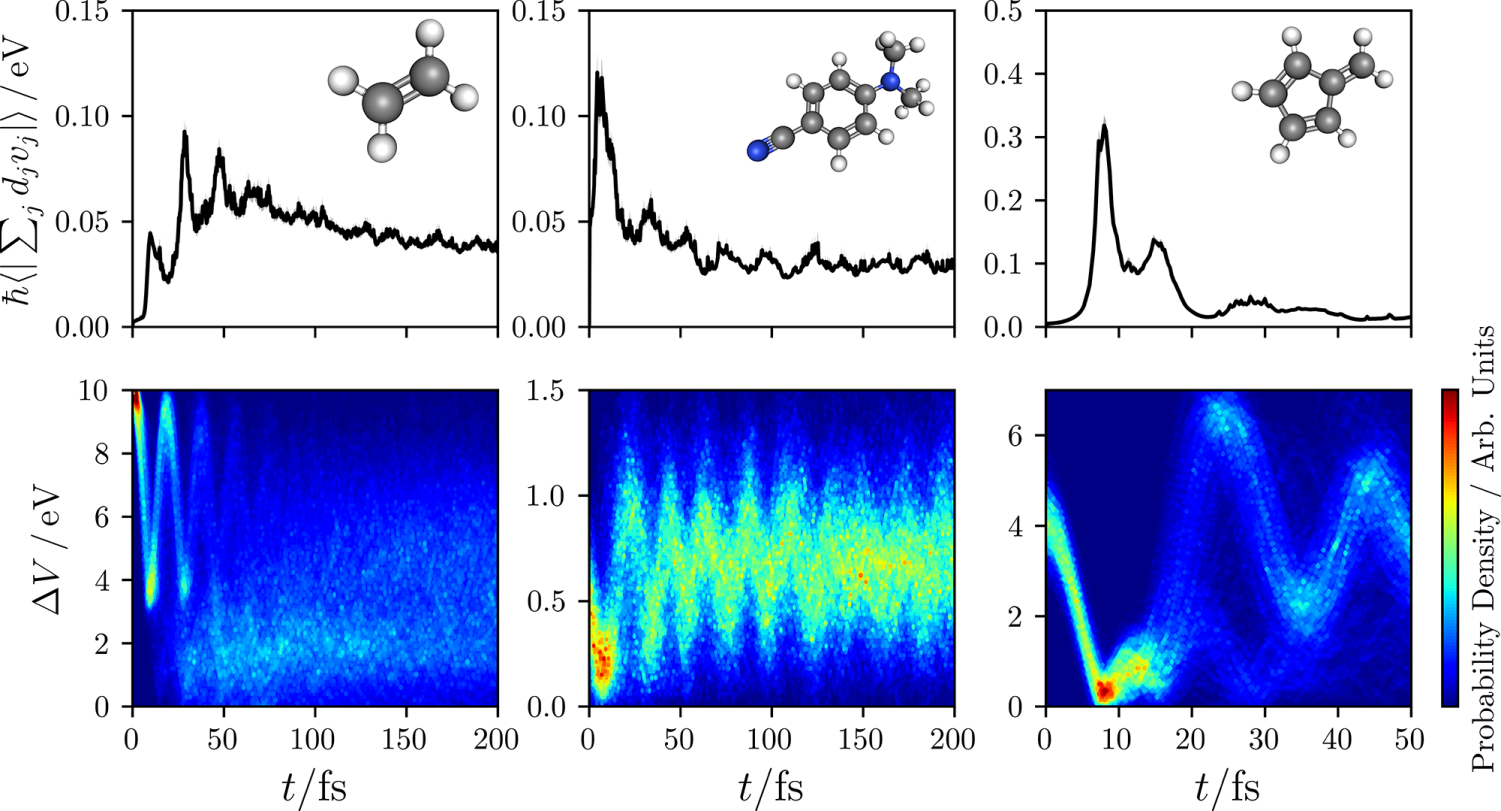
Magnitude of the effective nonadiabatic coupling, |*∑*_*j*_*d*_*j*_*v*_*j*_|, and the probability distribution of the time-dependent energy gap, Δ*V* = *V*_+_ – *V*_–_, between the two adiabatic states. These quantities are calculated for each system by averaging over the MASH trajectories.

Ethylene is known to have “indirect” access to its conical intersections,^7^ meaning that the Franck–Condon region for the S_0_ → S_1_ transition lies far away from the conical intersection seams and the initial nuclear dynamics upon photoexcitation does not directly access them. A redistribution of the vibrational energy to the appropriate modes during a few vibrational time periods is necessary before the crossing region can be accessed, giving a delayed onset of the first nonadiabatic transitions to ≈25 fs. At later times, the majority of the trajectories move away from the intersection region once they have reached the ground state. However, the relatively slow decay in the coupling suggests that this process is relatively inefficient and that many trajectories may undergo multiple nonadiabatic transitions before remaining on the ground-state surface.

In contrast, upon a Franck–Condon type photoexcitation from the ground state to S_2_, DMABN has a very small energy gap between S_2_ and S_1_. As a result, the nonequilibrium dynamics in DMBAN were previously coined “immediate”^7^ because the initial nuclear wavepacket is essentially on top of the conical intersection. The small initial energy gap between S_2_ and S_1_ also means that the dynamics remain close to the conical intersection seam for relatively long times, leading to the possibility that a large number of nonadiabatic transitions take place. This is consistent with the observation that the effective nonadiabatic coupling rapidly plateaus to a nonzero value. Experimentally, it is known that relaxation to the S_0_ state occurs at much longer times predominantly through fluorescence, and so we exclude the possibility of nonadiabatic transitions between S_1_ and S_0_ in this study.

Despite the conical intersection seams in fulvene being further away from the Franck–Condon region than in DMABN, the initial motion of the nuclear wavepacket does still allow direct access to the crossing region,^7^ in contrast to ethylene. The distinct feature of fulvene is that trajectories first pass through a sloped conical intersection seam driven by a stretch in the C=CH_2_ moiety,^5^ and then part of the wavepacket is reflected back through the crossing region at ≈15 fs. This system therefore provides a useful test for how well different nonadiabatic dynamics approaches can correctly describe recrossing phenomena. While a peaked conical intersection seam also exists in fulvene, driven by a twist in the C=CH_2_ moiety, we find that this intersection is not accessed until much later times.

We next analyze the time evolution of the electronic excited state populations, shown in the first row of Figure 2. In order to help analyze the differences in the obtained populations from different surface hopping algorithms, the number of allowed and frustrated hops is also given in the same figure. In particular, the adiabatic populations can be exactly reproduced from the difference in the number of downward and upward hops. Despite the differing properties of the conical intersections involved in these three systems, the general trend in the results for each method is largely the same.

**Figure 2:**
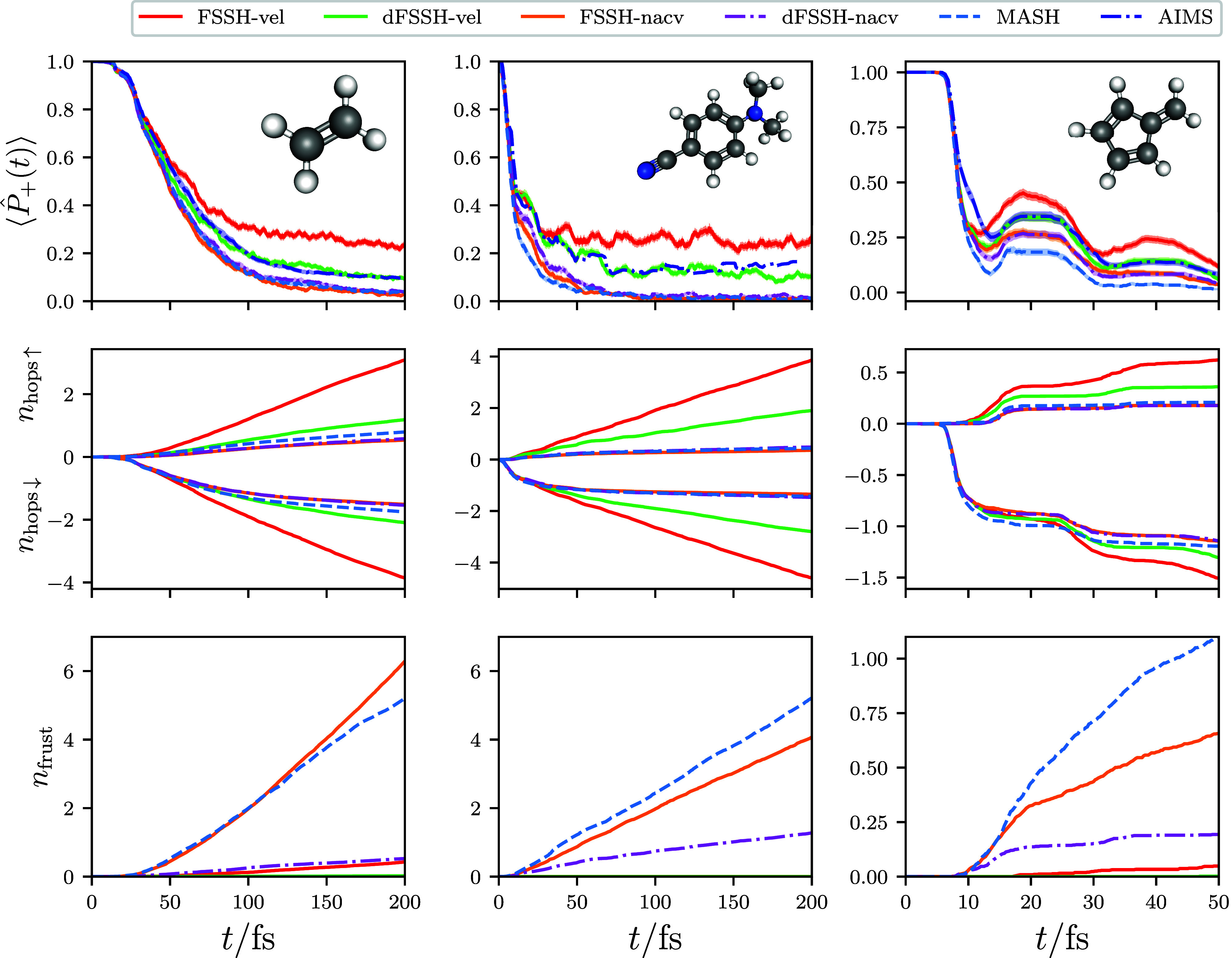
Electronic population of the upper adiabatic state  for ethylene, DMABN, and fulvene, computed for a wide variety of different methods. The width of the shading represents twice the standard error, which is less than the line width if not visible. The AIMS result for DMABN was obtained from ref (56). For the surface hopping approaches, the average number of upward hops (*n*_hops*↑*_) downward hops (*n*_hops*↓*_), and frustrated hops (*n*_frust_) are also given.

First, we observe that the direction in which the velocity rescaling is performed in surface hopping approaches results in a significant quantitative difference in the obtained electronic populations. For FSSH performed by rescaling along the velocity vector, dFSSH-vel deviates significantly from FSSH-vel, suggesting that the decoherence error in this case is large. In contrast, for the surface hopping results where the velocity rescaling is applied along the NACV, FSSH-nacv, and dFSSH-nacv are almost identical in all cases. This can be understood from the fact that, in all systems, FSSH-vel trajectories undergo a larger number of allowed hops than FSSH-nacv, making it more likely that the electronic wave function can become inconsistent with the active surface in the former. Decoherence corrections are therefore more necessary in FSSH-vel for reimposing consistency.

These differences between the algorithms can be further explained by considering the amount of nuclear kinetic energy available for promoting upward hops. In the case of rescaling along the velocity vector, the nuclear kinetic energy of the entire molecule is available for inducing electronic transitions, making almost all attempted hops energetically allowed. However, this behavior is unphysical because not all of the nuclear degrees of freedom are directly coupled to the electronic transition.^57,58^ In contrast, the NACV rescaling direction ensures that only the kinetic energy associated with directly coupled modes is considered; this is significantly less than the nuclear kinetic energy of the entire molecule, making upward hops more likely to be energetically forbidden and therefore frustrated. The associated electronic population for the upper adiabat is therefore significantly lower for dFSSH-nacv than for dFSSH-vel. This effect has been observed in other theoretical studies,^59^ including those on ethylene^60^ and fulvene.^5,27^

In particular, this leads to qualitatively different dynamics than was previously predicted for DMABN. One of the main reasons that DMABN was previously suggested as a good candidate benchmark system was that its dynamics were expected to produce similar features to Tully’s model II.^5^ It was known that the adiabatic potential energy surfaces remain close in energy throughout the dynamics (as also illustrated in Figure 1), suggesting that repeated electronic transitions between the surfaces would occur. While this is indeed observed when rescaling along the velocity vector, the dynamics observed when rescaling along the NACV instead involves a single rapid transition to the lower adiabatic state, where the system remains indefinitely. While the potential energy surfaces in DMABN do remain close together in energy relative to the kinetic energy of the entire molecule, they do not remain close together relative to the kinetic energy along the NACV.

Of the three systems, fulvene is particularly interesting because the MASH result significantly deviates from both FSSH-nacv and dFSSH-nacv. There is also a noticeable difference between these results for DMABN too, although the difference is much smaller. Figure 2 shows that this deviation between the MASH and dFSSH-nacv electronic populations is predominantly due to the larger number of frustrated hops in MASH, which leads to the MASH electronic populations being slightly lower than those of dFSSH-nacv.

What is also interesting about Figure 2 is that the electronic populations obtained by AIMS are seen to be almost identical with those obtained with dFSSH-vel. First, this suggests that the independent-trajectory approximation that underpins all surface hopping approaches is valid for these systems. While there are small deviations between the dFSSH-vel and AIMS results around 10 fs in fulvene and toward longer times in DMABN, we note that these differences are relatively minor compared to the more significant discrepancies observed between other algorithms. More importantly, the fact that AIMS and dFSSH-vel agree so well suggests that AIMS may not be describing the effect of the nonadiabatic force, which is an effect that is also neglected in dFSSH-vel. This finding is not so surprising in the case of DMABN, where the AIMS simulation did rescale the average velocity of spawned Gaussians along the velocity vector to ensure classical energy conservation.^56^ However, in the AIMS simulations for ethylene and fulvene, this rescaling was performed along the NACV, which at least for surface hopping algorithms is sufficient to correctly describe the nonadiabatic force. Our findings are, however, consistent with the observation that, unlike for surface hopping approaches, the velocity rescaling direction in AIMS is found to make almost no difference to the obtained results.^61^ While this point certainly requires further investigation, it does, however, suggest that the AIMS population dynamics may be less accurate than the best surface hopping algorithms in these cases.

In previous work, other algorithms have also been tested on these systems using the same initial conditions and electronic structure methods. In Figure S4 in the Supporting Information, we compare AIMS and MASH to various flavours of the symmetrical quasi-classical (SQC)^62^ method for the population dynamics of ethylene. The SQC results were obtained from ref (8). With the exception of non-gamma-corrected SQC using square windows, all of the other SQC approaches give results somewhere between AIMS and MASH. Given that the SQC results contain large statistical error, it is, however, hard to precisely ascertain the relative accuracies of the various approaches. While the surface hopping algorithms were performed with slightly more trajectories (1000) than the SQC approaches (500), given the observed noise in the data, we estimate that at least an order of magnitude more SQC trajectories would be needed to obtain a level of convergence similar to that of the surface hopping results. This highlights one of the main advantages of the surface hopping approaches (including MASH) over mean-field mapping-based approaches in that they require significantly fewer trajectories to converge the results.

To complement the above comparison of the various dynamics approaches in the *ab initio* case, we also performed simulations for linear vibronic coupling (LVC) models fit to the electronic structure data for DMABN and fulvene. These LVC models have already been used to perform numerically exact quantum dynamics using the multiconfiguration time-dependent Hartree (MCTDH) approach, along with some vMCG calculations.^7^ Given that it is significantly easier to compute diabatic populations with MCTDH, and adiabatic populations with AIMS, we provide both quantities in Figure 3. More details regarding the LVC model calculations can be found in Supporting Information. For the diabatic populations in the DMABN model, vMCG and MCTDH are essentially indistinguishable from one another and MASH, FSSH-nacv and dFSSH-nacv produce significantly more accurate results than FSSH-vel and dFSSH-vel. While the situation is less clear-cut in the fulvene model, both MASH and vMCG very accurately match the MCTDH result up to ≈10 fs and remain relatively close to it after that. For the adiabatic populations, the trend in the results for all of the approaches is largely the same as those in the *ab initio* simulations.^a^ Most importantly, the vMCG adiabatic populations agree best with those from MASH and FSSH-nacv, further suggesting that these surface hopping approaches are performing the best among all of the “on-the-fly” approaches that we have tested. The fact that the main difference between the vMCG and AIMS algorithms is how the Gaussians are propagated further suggests that this is the source of the error observed in the AIMS results.

**Figure 3:**
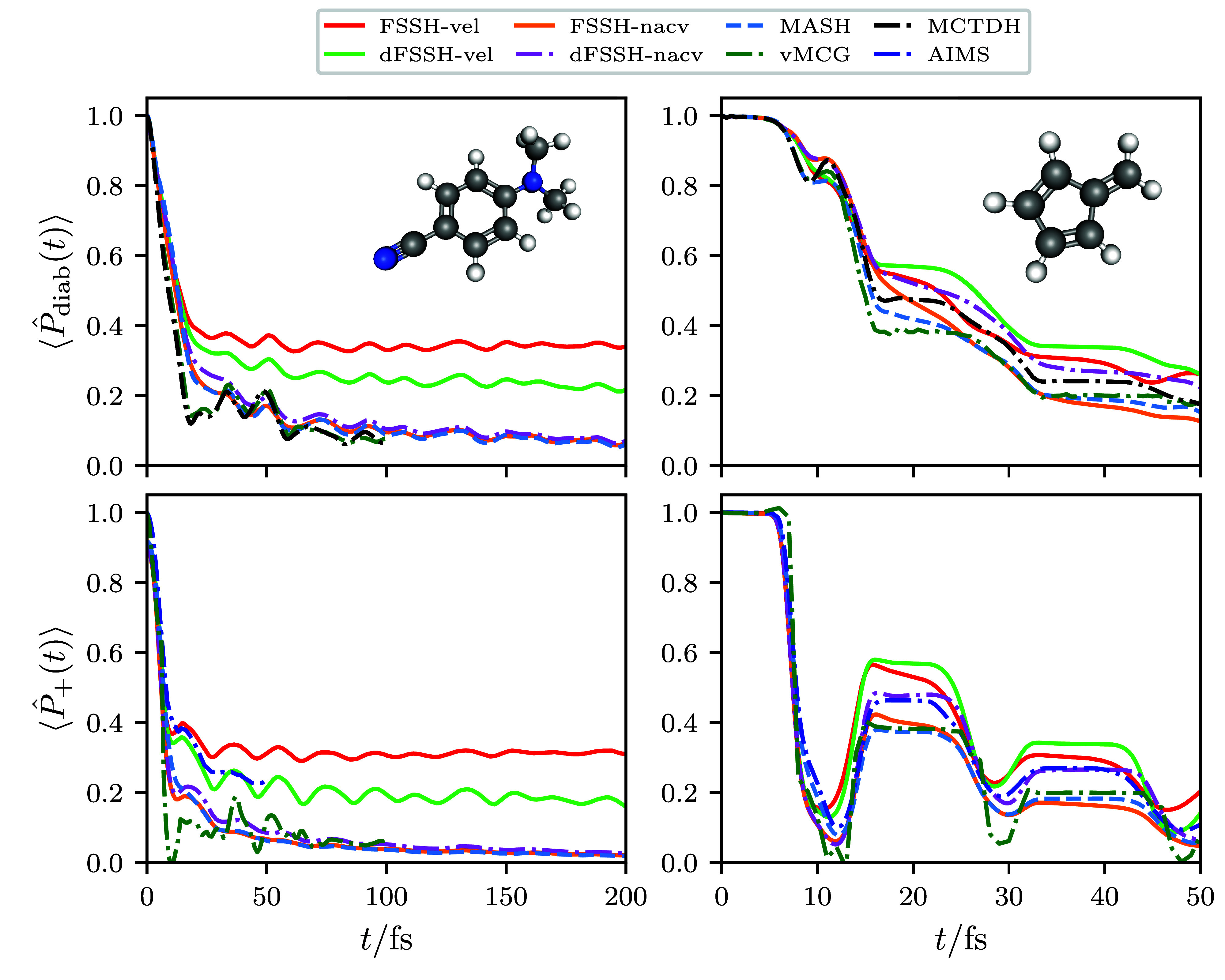
Electronic populations of the upper adiabatic state  and the diabatic state that coincides with this adiabat at the Franck–Condon geometry , obtained for the linear vibronic coupling (LVC) models that were constructed for DMABN and fulvene in ref (7). The MCTDH and vMCG results were also obtained from ref (7). We do not consider the analogous LVC model for ethylene, as it was found to not give rise to any electronic population transfer.

We now focus on comparing the best surface hopping approaches of FSSH-nacv, dFSSH-nacv, and MASH with AIMS for nuclear observables, and we provide the FSSH-vel and dFSSH-vel results in the Supporting Information for completeness. Not all nuclear observables are particularly sensitive to the nonadiabaticity of the problem, however. For example, one particularly interesting nuclear observable in the case of DMABN is the twist angle of the dimethylamino group. This is because the ground state structure is untwisted, while both of the minimum energy configurations of the S_1_ surface are twisted. In addition, there are two minimum energy conical intersections (MECIs) between S_1_ and S_2_, one of which is twisted and the other that is not.^56^ It is therefore interesting to ask whether the onset of twisting in the dynamics is directly connected to the nonadiabatic transition. In Figure S3 in the Supporting Information, we give the dynamical twist angle computed using a selection of methods. The fact that the observed twist angles are within the statistical error for all methods suggests that the nonadiabatic transitions are occurring through the untwisted MECI and that the observed twisting arises from the topology of the S_1_ surface. This is also in agreement with the conclusions of previous wavepacket and surface hopping simulations on DMABN.^56,63−65^

The product yields in the photodissociation of ethylene are an example of nuclear observables that end up being more sensitive to the nonadiabaticity of the problem. In the following, we group the possible products according to the four different channels depicted in Figure 4.^66,67^ All products observed in our computational simulations match those found in the corresponding experiments,^68−72^ with the exception of the C–C dissociation process. As commented on in previous theoretical studies of ethylene,^67^ the C–C dissociation is a result of the inadequacy of the basis set used in the electronic structure calculations, which gives a S_0_–S_1_ excitation energy of 10.2 eV at the Franck–Condon geometry, which is much larger than the experimental value of 7.6 eV^73^ and, most importantly, is significantly above the C–C bond energy of 7.7 eV.

**Figure 4:**
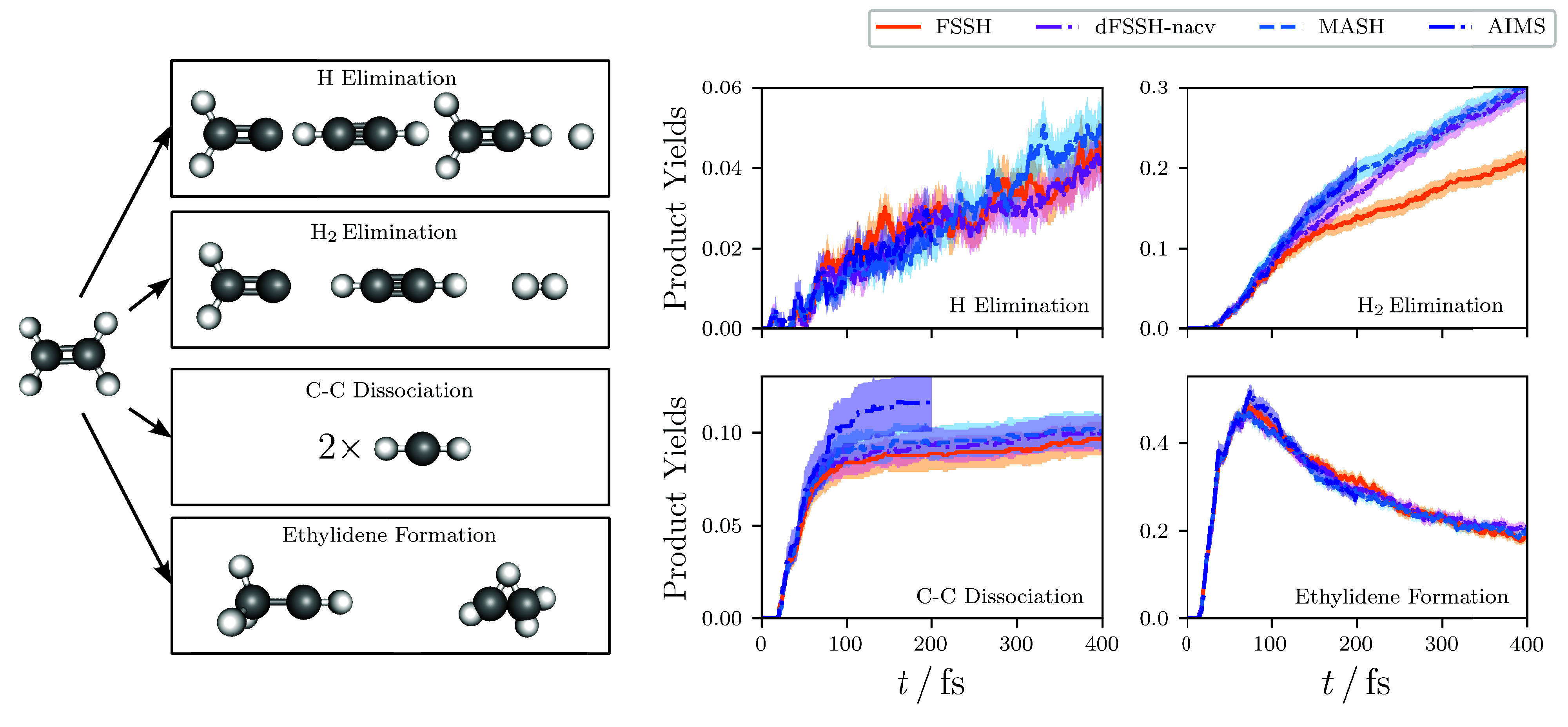
Dynamical product yields for the major products in the photodissociation of ethylene, calculated using various dynamical methods. The width of the shading represents twice the standard error.

The dynamical product yields calculated for a range of dynamical methods are listed in Figure 4. Of the products considered, the yields of H elimination, C–C dissociation, and ethylidene formation are largely the same for all the methods considered, at least within the statistical error. The geometries corresponding to ethylidene are known to match those of various conical intersection seams in the system.^67,74^ The product yield for this process therefore qualitatively describes the initial approach and subsequent exit of the conical intersection regions during the dynamics, explaining why it also qualitatively matches the average effective coupling between the Born–Oppenheimer surfaces, as given in Figure 1.

Of particular interest is the product yield for H_2_ elimination, where dFSSH-nacv, MASH, and AIMS all give rise to an enhanced product yield over FSSH-nacv. Such behavior is reminiscent of the use of surface hopping to calculate nonadiabatic thermal rates. In this case, the inconsistency error of FSSH is known to suppress the observed reaction rate and mean that the correct Δ^2^ scaling behavior with respect to the diabatic coupling strength is not correctly reproduced.^33,39−42^ More specifically in the thermal rate problem, it was found that, at short times, the two-hop trajectories between the ground-state reactant and product geometries were the ones that were responsible for the incorrectly suppressed reaction rate in FSSH.^38^ In the H_2_ elimination process, the trajectories associated with an odd number of hops are important because the reaction proceeds from the excited to the ground electronic state. As a result, we find that it is the three- and five-hop trajectories^b^ that are the problem for FSSH. The advantage of AIMS and MASH is that the correct nonadiabatic rate is reproduced without the need for ad hoc decoherence corrections. Note that, while performing AIMS simulations for long enough times to observe the reaction is expensive,^c^ this is not the case for the independent MASH trajectories.

In conclusion, we have applied a recently proposed independent-trajectory surface hopping approach, MASH, to perform *ab initio* nonadiabatic dynamics simulations in molecules. We compared MASH with a set of well established methods over a series of two-state benchmark systems, ethylene, DMABN, and fulvene, with the surfaces and couplings computed on-the-fly using various *ab initio* electronic structure methods. Both electronic and nuclear observables were considered.

Overall, MASH is likely to be the most suitable approach for performing *ab initio* simulations in molecules due to its accuracy and efficiency. For electronic population observables, MASH is able to correctly describe the effects arising from the nonadiabatic force, which was seen to be absent in AIMS and the most commonly used velocity rescaling scheme of FSSH. Such findings were also corroborated by the use of LVC models that were parametrized by fitting to electronic structure data for these molecules, where a comparison to numerically exact quantum dynamics was possible. Photodissociation product yields can also be accurately and robustly calculated with MASH, because it solves the inconsistency problem of FSSH without the need for ad hoc decoherence corrections.

Our analysis has uncovered a potential shortcoming within AIMS and it will be interesting to ascertain whether the nonadiabatic force can be correctly incorporated into the motion of the Gaussian basis functions within the AIMS algorithm. For the systems that we have tested here, the independent-trajectory approximation is seen to be valid, so that even if AIMS can be fixed, the computational simplicity of the independent-trajectory nature of MASH is still likely to be superior. An interesting question is therefore whether photochemical (or other molecular) processes can be found where the independent-trajectory approximation does break down and where coupled-trajectory approaches such as AIMS do offer a distinct advantage.

There are some approximations that are shared by all of the “on-the-fly” approaches tested in this work. For example, all of them sample the initial nuclear-phase-space variables from a Wigner distribution and propagate them classically. Therefore, it is hard to ascertain how severe the potential issues of zero-point energy leakage and the lack of nuclear tunneling are for these systems. MASH is guaranteed to thermalize correctly in the long-time limit with a classical nuclear bath,^76^ but this is not guaranteed if some of the nuclei have a large zero-point energy. A direct dynamics version of vMCG has been used to study these systems, and it would be good to estimate the robustness of the classical nuclear approximation in these systems by comparing our results to this in future work.

One problem of comparing different methods in *ab initio* simulations is the difficulty in making sure that the results obtained with different code bases are compatible with one another. To this end, we have provided an extensive Supporting Information which addresses in detail the potential sources of discrepancies between different codes, as well as showing how the dynamics in SHARC^52^ and the AIMS/MOLPRO package^55^ can be made consistent with one another. For future benchmarking exercises, it would be nevertheless useful to have more unified code packages where the majority of the most commonly used methods are implemented, as well as electronic structure codes where all possible quantities, such as NACVs,^77^ can be calculated.

The MASH algorithm used in this work is currently only applicable to two-state systems, and there is a need to generalize for multistate problems. Two distinct approaches have emerged for generalizing MASH,^78−80^ both of which were applied to study the photochemistry of cyclobutanone in the recent *J. Chem. Phys.* community challenge.^81,82^ We await to see how both ideas develop in the future.

## Supporting Information Available

Necessary information to reproduce the surface hopping and AIMS results in the main text including details on the electronic structure and dynamics methods employed, choosing the initial conditions for the simulations, constructing the nuclear observables for the product yields, MOLPRO input and SHARC template files, the ground-state geometries and frequencies, a python script for computing the ethylene product yields (ZIP)

## Footnotes

a Interestingly, the fulvene model unlike in the *ab initio* simulations seems to also provide a case for which applying decoherence corrections appears to make the FSSH results worse.

b In analogy to the thermal rate problem,^38^ one may expect that only the one- and three-hop trajectories are important for determining the rate. The reason that the 5-hop trajectories must also be considered is because the transient behavior is now longer as a result of this being a nonequilibrium rate process.

c Because of this issue, we only run the AIMS simulations up to 200 fs. Some algorithmic advancements in AIMS have been designed to alleviate this issue,^75^ but we do not directly consider them here.

## References

[ref1] Gao X.; Saller M. A. C.; Liu Y.; Kelly A.; Richardson J. O.; Geva E. Benchmarking Quasiclassical Mapping Hamiltonian Methods for Simulating Electronically Nonadiabatic Molecular Dynamics. J. Chem. Theory Comput. 2020, 16, 2883–2895. 10.1021/acs.jctc.9b01267. 32227993

[ref2] Runeson J. E.; Richardson J. O. Spin-mapping approach for nonadiabatic molecular dynamics. J. Chem. Phys. 2019, 151, 044119 10.1063/1.5100506. 31370524

[ref3] Runeson J. E.; Richardson J. O. Generalized spin mapping for quantum-classical dynamics. J. Chem. Phys. 2020, 152, 084110 10.1063/1.5143412. 32113368

[ref4] Mannouch J. R.; Richardson J. O. A partially linearized spin-mapping approach for nonadiabatic dynamics. I. Derivation of the theory. J. Chem. Phys. 2020, 153, 194109 10.1063/5.0031168. 33218231

[ref5] Ibele L. M.; Curchod B. F. E. A molecular perspective on Tully models for nonadiabatic dynamics. Phys. Chem. Chem. Phys. 2020, 22, 15183–15196. 10.1039/D0CP01353F. 32582887

[ref6] Tully J. C. Molecular dynamics with electronic transitions. J. Chem. Phys. 1990, 93, 1061–1071. 10.1063/1.459170.

[ref7] Gómez S.; Spinlove E.; Worth G. Benchmarking non-adiabatic quantum dynamics using the molecular Tully models. Phys. Chem. Chem. Phys. 2024, 26, 1829–1844. 10.1039/D3CP03964A. 38170796

[ref8] Weight B. M.; Mandal A.; Huo P. Ab initio symmetric quasi-classical approach to investigate molecular Tully models. J. Chem. Phys. 2021, 155, 084106 10.1063/5.0061934. 34470343

[ref9] Mannouch J. R.; Richardson J. O. A mapping approach to surface hopping. J. Chem. Phys. 2023, 158, 104111 10.1063/5.0139734. 36922129

[ref10] Subotnik J. E.; Jain A.; Landry B.; Petit A.; Ouyang W.; Bellonzi N. Understanding the surface hopping view of electronic transitions and decoherence. Annu. Rev. Phys. Chem. 2016, 67, 387–417. 10.1146/annurev-physchem-040215-112245. 27215818

[ref11] Ben-Nun M.; Quenneville J.; Martínez T. J. Ab Initio Multiple Spawning: Photochemistry from First Principles Quantum Molecular Dynamics. J. Phys. Chem. A 2000, 104, 5161–5175. 10.1021/jp994174i.

[ref12] Curchod B. F. E.; Martínez T. J. Ab Initio Nonadiabatic Quantum Molecular Dynamics. Chem. Rev. 2018, 118, 3305–3336. 10.1021/acs.chemrev.7b00423. 29465231

[ref13] Hammes-Schiffer S.; Tully J. C. Proton transfer in solution: Molecular dynamics with quantum transitions. J. Chem. Phys. 1994, 101, 4657–4667. 10.1063/1.467455.

[ref14] Bittner E. R.; Rossky P. J. Quantum decoherence in mixed quantum-classical systems: Nonadiabatic processes. J. Chem. Phys. 1995, 103, 8130–8143. 10.1063/1.470177.

[ref15] Jasper A. W.; Truhlar D. G. Electronic decoherence time for non-Born-Oppenheimer trajectories. J. Chem. Phys. 2005, 123, 064103 10.1063/1.1995695. 16122296

[ref16] Granucci G.; Persico M.; Zoccante A. Including quantum decoherence in surface hopping. J. Chem. Phys. 2010, 133, 134111 10.1063/1.3489004. 20942527

[ref17] Subotnik J. E.; Shenvi N. A new approach to decoherence and momentum rescaling in the surface hopping algorithm. J. Chem. Phys. 2011, 134, 024105 10.1063/1.3506779. 21241078

[ref18] Shenvi N.; Subotnik J. E.; Yang W. Simultaneous-trajectory surface hopping: A parameter-free algorithm for implementing decoherence in nonadiabatic dynamics. J. Chem. Phys. 2011, 134, 144102 10.1063/1.3575588. 21495737

[ref19] Subotnik J. E. Fewest-Switches Surface Hopping and Decoherence in Multiple Dimensions. J. Phys. Chem. A 2011, 115, 12083–12096. 10.1021/jp206557h. 21995423

[ref20] Jaeger H. M.; Fischer S.; Prezhdo O. V. Decoherence-induced surface hopping. J. Chem. Phys. 2012, 137, 22A545 10.1063/1.4757100. 23249082

[ref21] Vindel-Zandbergen P.; Ibele L. M.; Ha J.-K.; Min S. K.; Curchod B. F. E.; Maitra N. T. Study of the Decoherence Correction Derived from the Exact Factorization Approach for Nonadiabatic Dynamics. J. Chem. Theory Comput. 2021, 17, 3852–3862. 10.1021/acs.jctc.1c00346. 34138553 PMC8280698

[ref22] Schwerdtfeger C. A.; Soudackov A. V.; Hammes-Schiffer S. Nonadiabatic dynamics of electron transfer in solution: Explicit and implicit solvent treatments that include multiple relaxation time scales. J. Chem. Phys. 2014, 140, 034113 10.1063/1.4855295. 25669369

[ref23] Kapral R. Surface hopping from the perspective of quantum–classical Liouville dynamics. Chem. Phys. 2016, 481, 77–83. 10.1016/j.chemphys.2016.05.016.

[ref24] Subotnik J. E.; Ouyang W.; Landry B. R. Can we derive Tully’s surface-hopping algorithm from the semiclassical quantum Liouville equation? Almost, but only with decoherence. J. Chem. Phys. 2013, 139, 214107 10.1063/1.4829856. 24320364

[ref25] Pechukas P. Time-dependent semiclassical scattering theory. II. Atomic collisions. Phys. Rev. 1969, 181, 174 10.1103/PhysRev.181.174.

[ref26] Herman M. F. Nonadiabatic semiclassical scattering. I. Analysis of generalized surface hopping procedures. J. Chem. Phys. 1984, 81, 754–763. 10.1063/1.447708.

[ref27] Toldo J. M.; Mattos R. S.; Pinheiro M. J.; Mukherjee S.; Barbatti M. Recommendations for Velocity Adjustment in Surface Hopping. J. Chem. Theory Comput. 2024, 20, 614–624. 10.1021/acs.jctc.3c01159. 38207213

[ref28] Plasser F.; Crespo-Otero R.; Pederzoli M.; Pittner J.; Lischka H.; Barbatti M. Surface Hopping Dynamics with Correlated Single-Reference Methods: 9H-Adenine as a Case Study. J. Chem. Theory Comput. 2014, 10, 1395–1405. 10.1021/ct4011079. 26580359

[ref29] Suchan J.; Janoš J.; Slavíček P. Pragmatic Approach to Photodynamics: Mixed Landau–Zener Surface Hopping with Intersystem Crossing. J. Chem. Theory Comput. 2020, 16, 5809–5820. 10.1021/acs.jctc.0c00512. 32687703

[ref30] Papineau T. V.; Jacquemin D.; Vacher M. Which Electronic Structure Method to Choose in Trajectory Surface Hopping Dynamics Simulations? Azomethane as a Case Study. J. Phys. Chem. Lett. 2024, 15, 636–643. 10.1021/acs.jpclett.3c03014. 38205955

[ref31] Müller U.; Stock G. Surface-hopping modeling of photoinduced relaxation dynamics on coupled potential-energy surfaces. J. Chem. Phys. 1997, 107, 6230–6245. 10.1063/1.474288.

[ref32] Jasper A. W.; Truhlar D. G. Improved treatment of momentum at classically forbidden electronic transitions in trajectory surface hopping calculations. Chem. Phys. Lett. 2003, 369, 60–67. 10.1016/S0009-2614(02)01990-5.

[ref33] Jain A.; Subotnik J. E. Surface hopping, transition state theory, and decoherence. II. Thermal rate constants and detailed balance. J. Chem. Phys. 2015, 143, 134107 10.1063/1.4930549. 26450292

[ref34] Fang J.-Y.; Hammes-Schiffer S. Improvement of the Internal Consistency in Trajectory Surface Hopping. J. Phys. Chem. A 1999, 103, 9399–9407. 10.1021/jp991602b.

[ref35] Jasper A. W.; Stechmann S. N.; Truhlar D. G. Fewest-switches with time uncertainty: A modified trajectory surface-hopping algorithm with better accuracy for classically forbidden electronic transitions. J. Chem. Phys. 2002, 116, 5424–5431. 10.1063/1.1453404.

[ref36] Meyer H.-D.; Miller W. H. A classical analog for electronic degrees of freedom in nonadiabatic collision processes. J. Chem. Phys. 1979, 70, 3214–3223. 10.1063/1.437910.

[ref37] Stock G.; Thoss M. Semiclassical description of nonadiabatic quantum dynamics. Phys. Rev. Lett. 1997, 78, 578–581. 10.1103/PhysRevLett.78.578. 11580635

[ref38] Lawrence J. E.; Mannouch J. R.; Richardson J. O. Recovering Marcus Theory Rates and Beyond without the Need for Decoherence Corrections: The Mapping Approach to Surface Hopping. J. Phys. Chem. Lett. 2024, 15, 707–716. 10.1021/acs.jpclett.3c03197. 38214476 PMC10823533

[ref39] Landry B. R.; Subotnik J. E. Communication: Standard surface hopping predicts incorrect scaling for Marcus’ golden-rule rate: The decoherence problem cannot be ignored. J. Chem. Phys. 2011, 135, 191101 10.1063/1.3663870. 22112058

[ref40] Landry B. R.; Subotnik J. E. How to recover Marcus theory with fewest switches surface hopping: Add just a touch of decoherence. J. Chem. Phys. 2012, 137, 22A513 10.1063/1.4733675. 23249050

[ref41] Jain A.; Herman M. F.; Ouyang W.; Subotnik J. E. Surface hopping, transition state theory and decoherence. I. Scattering theory and time-reversibility. J. Chem. Phys. 2015, 143, 134106 10.1063/1.4930548. 26450291

[ref42] Falk M. J.; Landry B. R.; Subotnik J. E. Can Surface Hopping sans Decoherence Recover Marcus Theory? Understanding the Role of Friction in a Surface Hopping View of Electron Transfer. J. Phys. Chem. B 2014, 118, 8108–8117. 10.1021/jp5011346. 24745794

[ref43] Cotton S. J.; Liang R.; Miller W. H. On the adiabatic representation of Meyer-Miller electronic-nuclear dynamics. J. Chem. Phys. 2017, 147, 064112 10.1063/1.4995301. 28810754

[ref44] Mannouch J. R.; Richardson J. O. A mapping approach to surface hopping. J. Chem. Phys. 2023, 158, 104111 10.1063/5.0139734. 36922129

[ref45] Amati G.; Mannouch J. R.; Richardson J. O. Detailed balance in mixed quantum–classical mapping approaches. J. Chem. Phys. 2023, 159, 214114 10.1063/5.0176291. 38054513

[ref46] Ben-Nun M.; Martínez T. J. Nonadiabatic molecular dynamics: Validation of the multiple spawning method for a multidimensional problem. J. Chem. Phys. 1998, 108, 7244–7257. 10.1063/1.476142.

[ref47] Mignolet B.; Curchod B. F. E. A walk through the approximations of ab initio multiple spawning. J. Chem. Phys. 2018, 148, 134110 10.1063/1.5022877. 29626896

[ref48] Richings G. W.; Polyak I.; Spinlove K. E.; Worth G. A.; Burghardt I.; Lasorne B. Quantum dynamics simulations using Gaussian wavepackets: the vMCG method. Int. Rev. Phys. Chem. 2015, 34, 269–308. 10.1080/0144235X.2015.1051354.

[ref49] Ibele L. M.; Curchod B. F. E. Dynamics near a conical intersection—A diabolical compromise for the approximations of ab initio multiple spawning. J. Chem. Phys. 2021, 155, 174119 10.1063/5.0071376. 34742188

[ref50] Werner H.-J.; Knowles P. J.; Knizia G.; Manby F. R.; Schütz M.; MOLPRO, version 2012.1, a package of ab initio programs; 2012; http://www.molpro.net.

[ref51] Frisch M. J.; Trucks G. W.; Schlegel H. B.; Scuseria G. E.; Robb M. A.; Cheeseman J. R.; Scalmani G.; Barone V.; Petersson G. A.; Nakatsuji H.; Li X.; Caricato M.; Marenich A. V.; Bloino J.; Janesko B. G.; Gomperts R.; Mennucci B.; Hratchian H. P.; Ortiz J. V.; Izmaylov A. F.; Sonnenberg J. L.; Williams-Young D.; Ding F.; Lipparini F.; Egidi F.; Goings J.; Peng B.; Petrone A.; Henderson T.; Ranasinghe D.; Zakrzewski V. G.; Gao J.; Rega N.; Zheng G.; Liang W.; Hada M.; Ehara M.; Toyota K.; Fukuda R.; Hasegawa J.; Ishida M.; Nakajima T.; Honda Y.; Kitao O.; Nakai H.; Vreven T.; Throssell K.; Montgomery J. A. Jr.; Peralta J. E.; Ogliaro F.; Bearpark M. J.; Heyd J. J.; Brothers E. N.; Kudin K. N.; Staroverov V. N.; Keith T. A.; Kobayashi R.; Normand J.; Raghavachari K.; Rendell A. P.; Burant J. C.; Iyengar S. S.; Tomasi J.; Cossi M.; Millam J. M.; Klene M.; Adamo C.; Cammi R.; Ochterski J. W.; Martin R. L.; Morokuma K.; Farkas O.; Foresman J. B.; Fox D. J. Gaussian 16, Revision C.01; Gaussian Inc.: Wallingford, CT, 2016.

[ref52] Mai S.; Richter M.; Heindl M.; Menger M. F. S. J.; Atkins A.; Ruckenbauer M.; Plasser F.; Oppel M.; Marquetand P.; Gonzáalez L. SHARC2.0: Surface Hopping Including Arbitrary Couplings — Program Package for Non-Adiabatic Dynamics; https://sharc-md.org/, 2018.

[ref53] Plasser F.; Ruckenbauer M.; Mai S.; Oppel M.; Marquetand P.; González L. Efficient and Flexible Computation of Many-Electron Wave Function Overlaps. J. Chem. Theory Comput. 2016, 12, 1207–1219. 10.1021/acs.jctc.5b01148. 26854874 PMC4785508

[ref54] Mai S.; Marquetand P.; González L. Nonadiabatic dynamics: The SHARC approach. WIREs Comput. Mol. Sci. 2018, 8, e1370 10.1002/wcms.1370. PMC6220962 30450129

[ref55] Levine B. G.; Coe J. D.; Virshup A. M.; Martínez T. J. Implementation of ab initio multiple spawning in the Molpro quantum chemistry package. Chem. Phys. 2008, 347, 3–16. 10.1016/j.chemphys.2008.01.014.

[ref56] Curchod B. F. E.; Sisto A.; Martínez T. J. Ab Initio Multiple Spawning Photochemical Dynamics of DMABN Using GPUs. J. Phys. Chem. A 2017, 121, 265–276. 10.1021/acs.jpca.6b09962. 27976899

[ref57] Carof A.; Giannini S.; Blumberger J. Detailed balance, internal consistency, and energy conservation in fragment orbital-based surface hopping. J. Chem. Phys. 2017, 147, 214113 10.1063/1.5003820. 29221382

[ref58] Braun G.; Borges J.; Itamar; Aquino A. J. A.; Lischka H.; Plasser F.; do Monte S. A.; Ventura E.; Mukherjee S.; Barbatti M. Non-Kasha fluorescence of pyrene emerges from a dynamic equilibrium between excited states. J. Chem. Phys. 2022, 157, 154305 10.1063/5.0113908. 36272808

[ref59] Plasser F.; Mai S.; Fumanal M.; Gindensperger E.; Daniel C.; González L. Strong Influence of Decoherence Corrections and Momentum Rescaling in Surface Hopping Dynamics of Transition Metal Complexes. J. Chem. Theory Comput. 2019, 15, 5031–5045. 10.1021/acs.jctc.9b00525. 31339716

[ref60] Barbatti M. Velocity Adjustment in Surface Hopping: Ethylene as a Case Study of the Maximum Error Caused by Direction Choice. J. Chem. Theory Comput. 2021, 17, 3010–3018. 10.1021/acs.jctc.1c00012. 33844922

[ref61] Ibele L. M. From low dimensions to full configuration space: Generalising models for nonadiabatic molecular dynamics. Thesis; Durham University, 2022; http://etheses.dur.ac.uk/14321/.

[ref62] Miller W. H.; Cotton S. J. Classical molecular dynamics simulation of electronically non-adiabatic processes. Faraday Discuss. 2016, 195, 9–30. 10.1039/C6FD00181E. 27828549

[ref63] Du L.; Lan Z. An On-the-Fly Surface-Hopping Program JADE for Nonadiabatic Molecular Dynamics of Polyatomic Systems: Implementation and Applications. J. Chem. Theory Comput. 2015, 11, 1360–1374. 10.1021/ct501106d. 26574348

[ref64] Kochman M. A.; Tajti A.; Morrison C. A.; Miller R. J. D. Early Events in the Nonadiabatic Relaxation Dynamics of 4-(N,N-Dimethylamino)benzonitrile. J. Chem. Theory Comput. 2015, 11, 1118–1128. 10.1021/ct5010609. 26579762

[ref65] Gómez S.; Soysal E. N.; Worth G. A. Micro-Solvated DMABN: Excited State Quantum Dynamics and Dual Fluorescence Spectra. Molecules 2021, 26, 7247 10.3390/molecules26237247. 34885829 PMC8658867

[ref66] Barbatti M.; Ruckenbauer M.; Lischka H. The photodynamics of ethylene: A surface-hopping study on structural aspects. J. Chem. Phys. 2005, 122, 174307 10.1063/1.1888573. 15910032

[ref67] Miyazaki K.; Ananth N. Nonadiabatic simulations of photoisomerization and dissociation in ethylene using ab initio classical trajectories. J. Chem. Phys. 2023, 159, 124110 10.1063/5.0163371. 38127384

[ref68] Balko B. A.; Zhang J.; Lee Y. T. Photodissociation of ethylene at 193 nm. J. Chem. Phys. 1992, 97, 935–942. 10.1063/1.463196.

[ref69] Lin J. J.; Wang C. C.; Lee Y. T.; Yang X. Site-specific dissociation dynamics of ethylene at 157 nm: Atomic and molecular hydrogen elimination. J. Chem. Phys. 2000, 113, 9668–9677. 10.1063/1.1321044.

[ref70] Lee S.-H.; Lee Y. T.; Yang X. Dynamics of photodissociation of ethylene and its isotopomers at 157 nm: Branching ratios and kinetic-energy distributions. J. Chem. Phys. 2004, 120, 10983–10991. 10.1063/1.1740711. 15268128

[ref71] Kosma K.; Trushin S. A.; Fuss W.; Schmid W. E. Ultrafast Dynamics and Coherent Oscillations in Ethylene and Ethylene-d4 Excited at 162 nm. J. Phys. Chem. A 2008, 112, 7514–7529. 10.1021/jp803548c. 18661929

[ref72] Allison T. K.; Tao H.; Glover W. J.; Wright T. W.; Stooke A. M.; Khurmi C.; van Tilborg J.; Liu Y.; Falcone R. W.; Martínez T. J.; Belkacem A. Ultrafast internal conversion in ethylene. II. Mechanisms and pathways for quenching and hydrogen elimination. J. Chem. Phys. 2012, 136, 124317 10.1063/1.3697760. 22462867

[ref73] Robin M. B. Higher Excited States of Polyatomic Molecules; Elsevier Inc., 1985; Vol. 3.

[ref74] Levine B. G.; Martínez T. J. Isomerization Through Conical Intersections. Annu. Rev. Physiol. 2007, 58, 613–634. 10.1146/annurev.physchem.57.032905.104612. 17291184

[ref75] Lassmann Y.; Curchod B. F. E. AIMSWISS—Ab initio multiple spawning with informed stochastic selections. J. Chem. Phys. 2021, 154, 211106 10.1063/5.0052118. 34240975

[ref76] Amati G.; Mannouch J. R.; Richardson J. O. Detailed balance in mixed quantum–classical mapping approaches. J. Chem. Phys. 2023, 159, 214114 10.1063/5.0176291. 38054513

[ref77] Zhao X.; Merritt I. C. D.; Lei R.; Shu Y.; Jacquemin D.; Zhang L.; Xu X.; Vacher M.; Truhlar D. G. Nonadiabatic Coupling in Trajectory Surface Hopping: Accurate Time Derivative Couplings by the Curvature-Driven Approximation. J. Chem. Theory Comput. 2023, 19, 6577–6588. 10.1021/acs.jctc.3c00813. 37772732

[ref78] Runeson J. E.; Manolopoulos D. E. A multi-state mapping approach to surface hopping. J. Chem. Phys. 2023, 159, 094115 10.1063/5.0158147. 37675848

[ref79] Runeson J. E.; Fay T. P.; Manolopoulos D. E. Exciton dynamics from the mapping approach to surface hopping: comparison with Förster and Redfield theories. Phys. Chem. Chem. Phys. 2024, 26, 4929–4938. 10.1039/D3CP05926J. 38265093 PMC10849040

[ref80] Lawrence J. E.; Mannouch J. R.; Richardson J. O. A Size-Consistent Multi-State Mapping Approach to Surface Hopping. arXiv.2403.10627 2024, 10.48550/arXiv.2403.10627. 38940540

[ref81] Lawrence J. E.; Ansari I. M.; Mannouch J. R.; Manae M. A.; Asnaashari K.; Kelly A.; Richardson J. O. A MASH simulation of the photoexcited dynamics of cyclobutanone. J. Chem. Phys. 2024, 160, 174306 10.1063/5.0203695. 38748021

[ref82] Hutton L.; Carrascosa A. M.; Prentice A. W.; Simmermacher M.; Runeson J. E.; Paterson M. J.; Kirrander A. Using a multistate Mapping Approach to Surface Hopping to predict the Ultrafast Electron Diffraction signal of gas-phase cyclobutanone. arXiv.2402.10195 2024, 10.48550/arXiv.2402.10195. 38814011

