# Supplementary material for: Quantum Quality with Classical Cost: Ab Initio Nonadiabatic Dynamics Simulations Using the Mapping Approach to Surface Hopping
Source: J Phys Chem Lett. 2024 May 23;15(22):5814–23. doi: 10.1021/acs.jpclett.4c00535 (PMC11163471; doi:10.1021/acs.jpclett.4c00535)
Supplement: Supplementary file 1 — jz4c00535_si_001.zip [file jz4c00535_si_001.zip › SI/SI.pdf]

Supporting Information: Quantum Quality with Classical Cost: Ab Initio Nonadiabatic Dynamics Simulations using the Mapping Approach to Surface Hopping

Jonathan R. Mannouch* and Aaron Kelly*

Hamburg Center for Ultrafast Imaging, Universität Hamburg and the Max Planck Institute for the Structure and Dynamics of Matter, Luruper Chaussee 149, 22761 Hamburg, Germany

E-mail: jonathan.mannouch@mpsd.mpg.de; aaron.kelly@mpsd.mpg.de

Here we provide all of the necessary information to reproduce the results in the main text, including details on the electronic structure and dynamics methods employed, choosing the initial conditions for the simulations, and constructing the nuclear observables for the product yields.

Electronic structure

For ethylene and fulvene, the exited states are computed at the SA-CASSCF level of theory performed in MOLPRO 2012[S1] with (2,2) and (6,6) active spaces respectively, which contain all of the π and π^* orbitals within the molecules. A 6-31G* basis set with spherical harmonic basis functions is used. For ethylene, the state-averaging procedure is performed over the three lowest energy singlet states, but the dynamics is only considered in the subspace of the lowest two states. While previous work on the three-state case has suggested that S_2 can play an important role in the initial dynamics, we choose to stick with the simplicity of the two-state description for benchmarking purposes.[S2]

For DMABN, the S_1 and S_2 surfaces are computed using LR-TDDFT within the Tamm-Dancoff approximation using the LC-PBE functional and the 6-31G basis set. The TDDFT calculations

were performed in GAUSSIAN 16[S3] and the range-separation parameter for the functional was set to 0.3 Bohr.

The SHARC template and AIMS/MOLPRO input files for each molecule, which uniquely specifies the electronic structure theory used, are also provided.

Nonadiabatic Coupling Vectors

For LR-TDDFT calculations in GAUSSIAN 16,[S3] analytic NACVs are currently unavailable. The electronic propagation is therefore performed in a local diabatic basis,[S4] which is also the default option within the SHARC[S5,S6] package. A 'crude adiabatic basis' is chosen, which is the adiabatic basis associated with the nuclear geometry at the beginning of each time step. For the nuclear time step between t and $t + \Delta t$, the electronic Hamiltonian at time t is diagonal in this basis, while the Hamiltonian at time $t + \Delta t$ can be expressed as a similarity transform of the associated potential energies,

$$H_{\beta\gamma}(\boldsymbol{q}(t)) = V_{\beta}(\boldsymbol{q}(t))\delta_{\beta\gamma}, \tag{S1a}$$

$$H_{\beta\gamma}(\boldsymbol{q}(t+\Delta t)) = \sum_{\alpha} S_{\beta\alpha} V_{\alpha}(\boldsymbol{q}(t+\Delta t)) S^{\dagger}_{\alpha\gamma}, \tag{S1b}$$

$$S_{\beta\alpha} = \langle \psi_{\beta}(\boldsymbol{q}(t)) | \psi_{\alpha}(\boldsymbol{q}(t+\Delta t)) \rangle \, \mathrm{sgn}(\langle \psi_{\alpha}(\boldsymbol{q}(t)) | \psi_{\alpha}(\boldsymbol{q}(t+\Delta t)) \rangle). \tag{S1c}$$

The sign function ensures that the wavefunctions at the two times have a consistent sign, with the overall sign of the overlap matrix given by the sign of the wavefunction at time t. The sign of the wavefunction at time $t + \Delta t$ is then updated to be consistent with the sign of the wavefunction at time t, in order to calculate the overlap matrix at the next time step.

For the electronic propagation across the nuclear time step, Δt, the propagation is further split up into n_{elec} electronic time steps, $\delta t = \Delta t / n_{\mathrm{elec}}$ and the electronic Hamiltonian is linearly interpolated between Eq. S1a and Eq. S1b. The propagation of the electronic wavefunction coefficients

is then given by

$$\tilde{H}_{\alpha\beta}(t_k) = H_{\alpha\beta}(\boldsymbol{q}(t)) + \frac{k}{n_{\text{elec}}}\left[H_{\alpha\beta}(\boldsymbol{q}(t+\Delta t)) - H_{\alpha\beta}(\boldsymbol{q}(t))\right] \tag{S2a}$$

$$\tilde{c}_\alpha(t+\Delta t) = \sum_\beta \left[\prod_{k=1}^{n_{\text{elec}}} \mathrm{e}^{-\frac{i}{\hbar}\tilde{\boldsymbol{H}}(t_k)\delta t}\right]_{\alpha\beta} \tilde{c}_\beta(t), \tag{S2b}$$

where $\mathrm{e}^{-i\tilde{\boldsymbol{H}}(t_k)}$ is a matrix exponential, $\tilde{c}_\alpha$ are the electronic wavefunction coefficients in the crude adiabatic basis and $\tilde{c}_\alpha(t) = c_\alpha(t)$. In this work, we use $n_{\text{elec}} = 25$. Once the electronic wavefunction has been successfully propagated to $t + \Delta t$, it is then transformed back into the adiabatic basis as follows

$$c_\alpha(t+\Delta t) = \sum_\beta S^\dagger_{\alpha\beta}\,\tilde{c}_\beta(t+\Delta t). \tag{S3}$$

Given that the couplings in the diabatized Hamiltonian are generally less localized compared to the NACVs, we use this electronic propagation scheme even when analytic NACVs are available.

In order to rescale the velocity when analytic NACVs are not available, we use the following finite difference scheme

$$\begin{aligned} d_j(\boldsymbol{q}) \quad &\approx \quad \frac{1}{2\Delta x}\Big(\langle\psi_+(\boldsymbol{q})|\psi_-(\boldsymbol{q}+\Delta x\boldsymbol{j})\rangle\,\mathrm{sgn}(\langle\psi_-(\boldsymbol{q})|\psi_-(\boldsymbol{q}+\Delta x\boldsymbol{j})\rangle) \\ &- \quad \langle\psi_+(\boldsymbol{q})|\psi_-(\boldsymbol{q}-\Delta x\boldsymbol{j})\rangle\,\mathrm{sgn}(\langle\psi_-(\boldsymbol{q})|\psi_-(\boldsymbol{q}-\Delta x\boldsymbol{j})\rangle)\Big) \end{aligned} \tag{S4}$$

where $\boldsymbol{j}$ is a unit vector pointing along the Cartesian coordinate associated with nuclear degree of freedom, j. Because the NACV is only used to determine the direction of the velocity rescaling, the signs of the NACVs at different time steps do not have to be consistent. The NACV is only computed at time steps where either a hop or frustrated hop have taken place and we use $\Delta x = 0.001$ Bohr in all of our simulations.

In order to test this finite difference scheme, $\sum_j d_j(\boldsymbol{q}(t))v_j(t)$ can be calculated in the small nuclear time step limit as

$$\sum_j d_j(\boldsymbol{q}(t))v_j(t) \approx \frac{1}{\Delta t}\,\langle\psi_+(\boldsymbol{q}(t))|\psi_-(\boldsymbol{q}(t+\Delta t))\rangle\,\mathrm{sgn}(\langle\psi_-(\boldsymbol{q}(t))|\psi_-(\boldsymbol{q}(t+\Delta t))\rangle), \tag{S5}$$

and compared with the same quantity computed using Eq. S4. Additionally, Eq. S5 was used to

calculate $\sum_j d_j(\boldsymbol{q}(t))v_j(t)$ for DMABN in Fig. 1 of the main paper.

For both the finite difference scheme for the NACVs and the local diabatic electronic propagation scheme, the required wavefunction overlaps were computed using the associated code in SHARC.[S4] In addition, a Löwdin orthogonalization is performed on all overlap matrices to ensure unitarity. In the case of ethylene, this orthogonalization procedure is performed in the three-state space.

Dynamics Methods

Ab Initio Multiple Spawning (AIMS)

Our AIMS simulations for ethylene and fulvene were performed using the AIMS/MOLPRO code.[S7] A time step of 20 a.u. was used outside the nonadiabatic coupling region. The coupling region is entered when $\hbar \sum_j d_j v_j > 0.005\, E_h$, for which the time step was reduced to 5 a.u.. The value of this coupling threshold parameter was determined by reducing its value until there was no noticeable change in the obtained results.

In the coupling region, Gaussians can be spawned whenever $\sum_j d_j v_j$ reaches a maximum along the trajectory. For this to occur, the overlap between the parent and child Gaussians at the spawning point must also exceed a threshold value, set for our calculations to 0.6. A minimum population of 0.01 is also required for a Gaussian to spawn a child.

The threshold for energy violation over a time step is set for ethylene and fulvene as 0.03 and 0.01 E_h respectively. If this is violated, the time step is halved unless the minimum value of 1 a.u. is reached, after which energy violation is ignored. Ad hoc features of the AIMS simulations, such as setting a decoherence time, are not used in our simulations in order to obtain the most accurate and rigorous AIMS benchmark for these systems.

When performing AIMS simulations, it is important that the initial nuclear geometry in the MOLPRO input and Geometry.dat files exactly match for each trajectory. The initial momenta in the Geometry.dat file must be provided in atomic units. This can be obtained from the initial velocities used in SHARC by multiplying them by the appropriate atomic mass in atomic units.

For the AIMS simulations, 400 and 150 trajectories were run for ethylene and fulvene respectively. Of these, 8 trajectories failed for ethylene due to a failure in converging the CASSCF cycle. We did not perform the AIMS calculation for DMABN ourselves and the results were instead taken

from Ref. S8. The details for the AIMS simulation of DMABN can therefore be found there.

For the linear vibronic coupling (LVC) models, the AIMS dynamics were performed with a modified version of the FMS90 code implemented in MOLPRO.[S9] 2000 AIMS trajectories were run with a times step of 2.0 a.u., reduced to 0.5 a.u. in the coupling region. The coupling region was entered when $\hbar \sum_j d_j v_j > 0.002\, E_h$ and a minimum population of 0.001 and an overlap threshold of 0.6 were set for spawning Gaussians. All other parameters were kept at their default value. For AIMS simulations, the system has to be initialized in an adiabatic state, whereas for all other approaches in the LVC models, we initialized the dynamics in a diabatic state. Fig. S1 gives the MASH adiabatic populations associated with starting in an adiabatic and diabatic state, which illustrates that apart from the $t = 0$ value in the DMABN model, the initial conditions do not make much of a difference to the obtained populations in these LVC models.

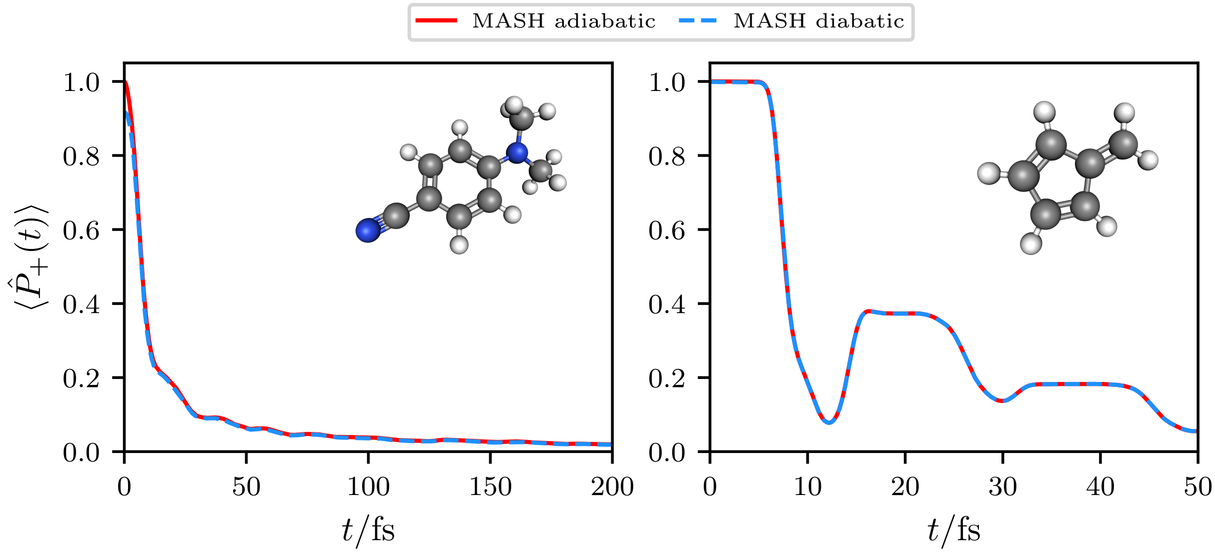

Figure S1: Figure showing the MASH dynamical populations of the upper adiabatic state in the LVC models, when starting in either an adiabatic or diabatic state. The MASH results when starting in an initial diabatic state are the same as given in Fig. 3 of the main paper.

As is standard practice in AIMS, the electronic populations are calculated using the overlaps between the Gaussians[S8]

$$\langle \hat{P}_+(t) \rangle = \frac{1}{N_{\mathrm{runs}}} \sum_{n=1}^{N_{\mathrm{runs}}} \sum_{k,l=1}^{N_{\mathrm{Gauss},n}(t)} C^*_{k,n,+}(t) C_{l,n,+}(t) \left\langle g_{k,n}(\boldsymbol{q}_{k,n}(t), \boldsymbol{p}_{k,n}(t)) | g(\boldsymbol{q}_{l,n}(t), \boldsymbol{p}_{l,n}(t)) \right\rangle , \quad \text{(S6)}$$

where N_{runs} is the number of independent AIMS runs and $N_{\mathrm{Gauss,n}}(t)$ is the number of Gaussians

in run n at time t. Additionally, $q_{k,n}(t)$ and $p_{k,n}(t)$ are the classical nuclear phase-space variables at which the Gaussian $g_{k,n}$ is centered and $C_{k,n,+}(t)$ is its time-dependent weight, where the + subscript indicates that only Gaussians on the upper surface are used to compute the population of the upper adiabat.

In contrast for nuclear observables like the DMABN twist angle and the ethylene product yields, only the diagonal elements of the Gaussian basis are used. For observables $\hat{O}$ that are functions solely of the nuclear positions, $\hat{\boldsymbol{q}}$[S8]

$$\langle \hat{O}(t) \rangle \approx \frac{1}{N_{\text{runs}}} \sum_{n=1}^{N_{\text{runs}}} \frac{\sum_{k=1}^{N_{\text{Gauss},n}} |C_{k,n}(t)|^2 \, O(\boldsymbol{q}_{k,n}(t))}{\sum_{k=1}^{N_{\text{Gauss},n}} |C_{k,n}(t)|^2}, \tag{S7}$$

where the lack of the + subscript on the Gaussian weights now indicates that all Gaussians contribute to the observable, irrespective of the surface they are on.

Fewest-Switches Surface Hopping (FSSH)

In FSSH,[S10] the nuclei are propagated according to Newton's equations of motion on the active Born-Oppenheimer surface and the electronic wavefunction is propagated according to the time-dependent Schrödinger equation [Eq. S2b]. The velocity-verlet scheme is used for the nuclear dynamics with $\Delta t = 0.25$ fs. In order to initialize the electronic subsystem in the excited state, the initial wavefunction coefficients are given by $c_-(0) = 0$ and $c_+(0) = 1$.

In order to describe nonadiabatic transitions, the active surface is changed stochastically with the following probability

$$P_{\alpha \to \beta} = \frac{2\text{Re}\left[c_\alpha^* S_{\alpha\beta} c_\beta\right]}{|c_\alpha|^2}, \tag{S8}$$

where Re[·] returns the real part of the quantity and any negative probabilities are set to zero. Electronic population observables are constructed in terms of the active state, rather than the electronic wavefunction coefficients.

At an attempted hop, the kinetic energy along the NACV must first be compared with the electronic transition energy. A hop from state α to β is energetically allowed if

$$\frac{\left(\sum_j d_j v_j\right)^2}{2\sum_j \frac{d_j^2}{m_j}} > V_\beta - V_\alpha, \tag{S9}$$

in which case the velocity along the NACV is rescaled according to

$$v_n^{\text{new}} = v_n^{\text{old}} - \frac{\frac{d_n}{m_n}}{\sum_j \frac{d_j^2}{m_j}} \left[\sum_j d_j v_j^{\text{old}} - \text{sgn}\left(\sum_j d_j v_j^{\text{old}} \right) \sqrt{\left(\sum_j d_j v_j^{\text{old}} \right)^2 + 2(V_\alpha - V_\beta) \left(\sum_j \frac{d_j^2}{m_j} \right)} \right]. \tag{S10}$$

In the event that Eq. S9 is not satisfied, the hop is frustrated and the nuclear velocity along the NACV is reflected as follows

$$v_n^{\text{new}} = v_n^{\text{old}} - 2 \left(\sum_j d_j v_j^{\text{old}} \right) \frac{\frac{d_n}{m_n}}{\sum_j \frac{d_j^2}{m_j}}. \tag{S11}$$

This treatment of frustrated hops in FSSH required a modification to the SHARC code in order to ensure that the nuclear velocity is always reflected at a frustrated hop.

To add decoherence into FSSH simulations, we use the energy-based decoherence scheme described in Ref. S11,S12. For a trajectory with current active surface β, the electronic coefficients are updated at each time step as follows

$$c_\alpha^{\text{new}} = c_\alpha^{\text{old}} \mathrm{e}^{-\frac{1}{2}\Delta t \frac{|V_\alpha - V_\beta|}{\hbar\left(1 + \frac{C}{E_{\text{kin}}}\right)}}, \tag{S12a}$$

$$c_\beta^{\text{new}} = \frac{c_\beta^{\text{old}}}{|c_\beta^{\text{old}}|} \sqrt{1 - \sum_{\alpha \neq \beta} |c_\alpha^{\text{new}}|^2}, \tag{S12b}$$

where E_{kin} is the total nuclear kinetic energy and $C = 0.1\, E_h$ is the decoherence parameter.

All FSSH simulations were performed using SHARC 2.0.[S6] For ethylene, DMABN and fulvene, 1000, 600 and 600 trajectories were run respectively to ensure well converged results. However far fewer trajectories would be needed for a good qualitative description of the observables. For ethylene only 5 trajectories or fewer failed as a result of being unable to converge the CASSCF cycle, whereas for fulvene it was 3 trajectories or fewer.

For the LVC models, the surface hopping approaches were initialized in a diabatic state, to allow direct comparison with the MCTDH and vMCG results. Ref. S13 explains how to compute diabatic populations with FSSH. We choose to use a very large number of trajectories (100000) to essentially remove any statistical error within the results, which is easy to do in model calculations. However, far fewer trajectories could have been used in practice to qualitatively reproduce the

correct dynamical behaviour.

A Mapping Approach to Surface Hopping (MASH)

In order to perform *ab initio* MASH simulations, a modified version of SHARC was used. Due to the similarities between the FSSH and MASH algorithms, only minor modifications to the existing surface-hopping routine were required, which we describe here.

The first difference between FSSH and MASH is the initial sampling of the electronic wavefunction. When starting in the upper adiabatic state, the electronic wavefunction in MASH is sampled from the upper hemisphere of the Bloch sphere.[S14] This corresponds to

$$c_+ = \cos\left(\frac{\theta}{2}\right)\mathrm{e}^{-i\phi/2}, \tag{S13a}$$

$$c_- = \sin\left(\frac{\theta}{2}\right)\mathrm{e}^{i\phi/2}, \tag{S13b}$$

with $0 \leq \theta < \pi/2$ and $0 \leq \phi < 2\pi$. The sampling over the upper hemisphere of the Bloch sphere is not uniform however, but is weighted by the factor: $|c_+|^2 - |c_-|^2$. To incorporate this, the values of θ and ϕ are sampled according to

$$\theta = \cos^{-1}(\sqrt{a}), \tag{S14a}$$

$$\phi = 2\pi b, \tag{S14b}$$

where a and b are themselves sampled uniformally and independently between the values 0 and 1. In SHARC, this can be easily implemented by sampling the initial electronic wavefunction externally and then reading it in through an external coefficient file.

The second difference is that in MASH, the active surface is no longer a stochastic variable, but is uniquely determined from the electronic wavefunction.[S14] The active surface variable, n_{active}, in MASH is given by

$$n_{\text{active}} = \mathrm{sgn}(|c_+|^2 - |c_-|^2), \tag{S15}$$

where $n_{\text{active}} = 1$ corresponds to propagation on the upper surface and $n_{\text{active}} = -1$ on the lower surface.

All MASH simulations were performed using a locally modified (as described above) version of SHARC 2.0.[S6] For ethylene, DMABN and fulvene, 1000, 600 and 600 trajectories were run respectively to ensure well converged results. However far fewer trajectories would be needed for a good qualitative description of the observables. For ethylene, only 4 trajectories failed as a result of being unable to converge the CASSCF cycle.

For the LVC models, MASH was initialized in a diabatic state, to allow direct comparison with the MCTDH and vMCG results. Ref. S13 explains how to do this with MASH, as well as how to compute diabatic populations. We choose to use a very large number of trajectories (100000) to essentially remove any statistical error within the results, which is easy to do in model calculations. However, far fewer trajectories could have been used in practice to qualitatively reproduce the correct dynamical behaviour.

Nuclear Initial Conditions Sampling

The nuclei were initialized in the nuclear ground-state associated with the ground-state Born-Oppenheimer surface. As is common practice, this was approximated as a multidimensional harmonic oscillator. The optimization of the ground-state geometry, q_j' and frequencies, ω_j, were performed using the same level of electronic structure as the dynamics, with the atomic masses chosen to be consistent with the AIMS/MOLPRO code.[S7] These are given in Table. S1 in both atomic units (a.u.) and atomic mass units (a.m.u.). For each system, the MOLDEN files containing the equilibrium ground-state geometry and frequencies are also provided.

Table S1: Atomic masses used for the *ab initio* simulations performed in this work, given in atomic units (a.u.) and atomic mass units (a.m.u.).

Atom	C	H	N
Mass (a.u.)	21874.644	1822.887	25520.418
Mass (a.m.u.)	11.999990224335	0.999999185361	13.999988595055

For the dynamical approaches considered in this work, the initial nuclear phase-space variables for the trajectories are sampled from the Wigner distribution of the ground-state quantum harmonic

oscillator

$$\rho_{\text{nuc}}(\tilde{\boldsymbol{q}}, \tilde{\boldsymbol{p}}) = \prod_{j=1}^{N} \frac{\hbar}{\pi} \exp\left[-\frac{\tilde{p}_j^2 + \omega_j^2 \left(\tilde{q}_j - \tilde{q}_j'\right)^2}{\hbar\omega_j} \right], \tag{S16}$$

where the tildes signify mass-weighted coordinates. In SHARC, this sampling can be performed using the command

```
$SHARC/wigner.py -n 600 -m freq.molden
```

where the -m option allows the use of non-standard masses, the -n option specifies the number of samples (in this case 600) and freq.molden is the MOLDEN file containing the frequencies and the ground-state geometry.

Not all previous simulations on these systems used the same nuclear initial conditions however. For the AIMS simulation of DMABN in Ref. S8, initial Wigner sampled phase-space variables were further refined according to their associated oscillator strength for the $S_0 \rightarrow S_2$ photoexcitation transition. For the surface-hopping algorithms, we tested the initial nuclear conditions with and without this further refinement and found that it only made negligible differences to the results. We therefore chose to present the results corresponding to sampling from the Wigner distribution given by Eq. S16.

Additionally in Ref. S15, the dynamical simulations of fulvene set the initial nuclear velocity to zero, in order to ensure that trajectories only initially passed through the sloped conical intersection seam. We however find that this is also the case when trajectories are sampled from the initial Wigner distribution, as well as having the additional advantage of corresponding to a valid quantum state.

Nuclear Observables

Product Yields in Ethylene

In order to define the various products arising from the photoexcitation of ethylene, certain bond lengths must first be defined. Most straightforwardly, the carbon-carbon bond length, $r_{\text{C}-\text{C}}$, is defined as the distance between the two carbon atoms, C_1 and C_2. For each hydrogen atom, H_i, the corresponding carbon-hydrogen bond length, $r_{\text{C}-\text{H}_i}$, is taken as the smallest of $r_{\text{C}_1-\text{H}_i}$

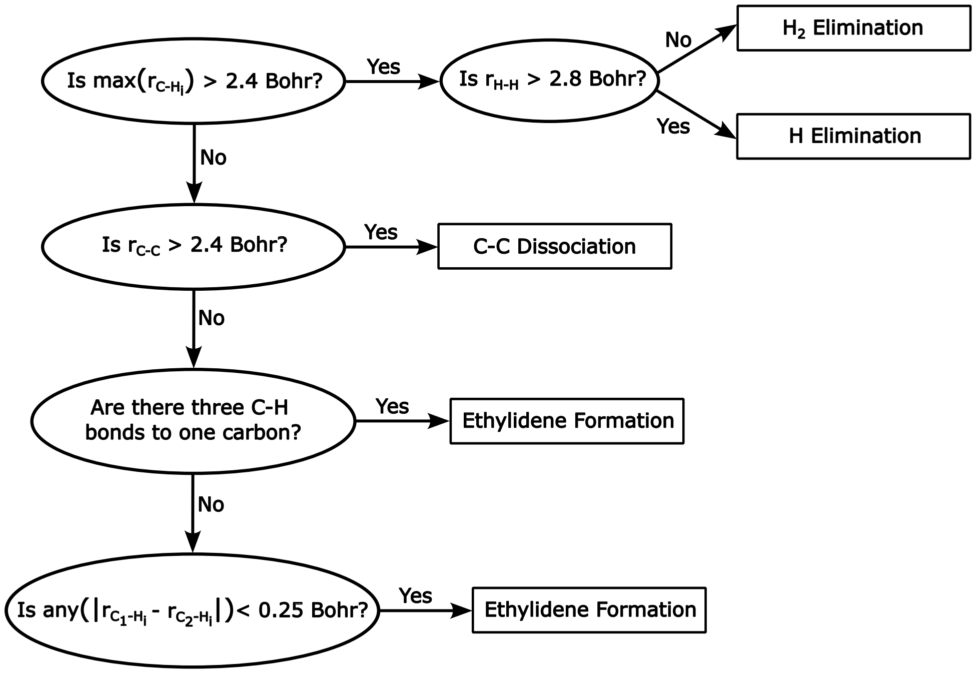

Figure S2: A flowchart defining the various products formed after the photoexcitation of ethylene. The first row of the diagram determines whether any C-H bonds have been cleaved, the second whether the C-C bond has been broken, the third whether any hydrogen atoms have migrated to another carbon and the fourth looks for any hydrogen bridging atoms.

and $r_{\mathrm{C}_2-\mathrm{H}_i}$. Finally, we define H_4 and H_3 as the two hydrogen atoms with the largest and second largest carbon-hydrogen bond lengths respectively (and are therefore the hydrogens that have either dissociated or are closest to doing so). The hydrogen-hydrogen bond distance, $r_{\mathrm{H-H}}$, is then defined as the distance between H_4 and H_3.

Figure. S2 contains a flowchart that shows how we determined the products formed at a given time step of a trajectory. This was based on the scheme used in Ref. S16 and was tested by analyzing a sufficiently large random selection of trajectories by hand. The bond-length criteria used in our product specifications are very different from the equilibrium bond lengths in ethylene, because ethylene is highly vibrationally excited once it relaxes back to the electronic ground state after the initial electronic photoexcitation.

The Twist Angle of the Dimethylamino Group in DMBAN

We use the same definition of the twist angle of the dimethylamino group in DMABN as Eq. 7 in Ref. S8. Figure S3 gives the twist angle of the dimethylamino group in DMABN as a function of

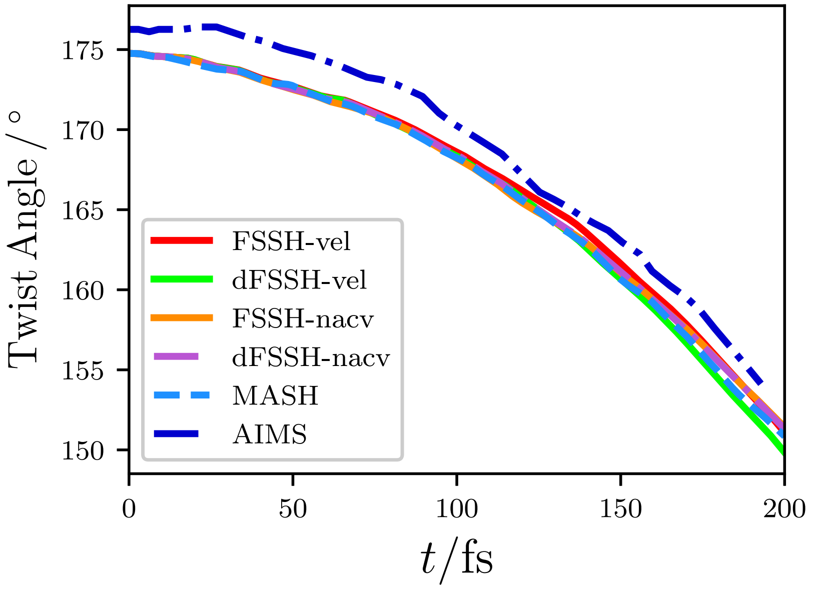

Figure S3: The twist angle of the dimethylamino group in DMABN. The AIMS result is taken from Ref. S8.

time. The AIMS result was taken from Ref. S8. Because only 20 AIMS trajectories were used to calculate the twist angle (compared to 200 trajectories for the surface hopping results), we expect that the small difference between the AIMS and surface hopping twist angles is due to statistical error in the former.

Additional results

In Fig. S4 we compare SQC results from Ref. S17 with our AIMS and MASH results for the excited state population of ethylene.

Additionally, Fig. S5 gives the dynamical product yields calculated for FSSH-vel and dFSSH-vel, which were not included in the main paper. Interestingly unlike FSSH-nacv, FSSH-vel is able to accurately reproduce the product yield for H_2 elimination, but instead under predicts the product yield for H elimination.

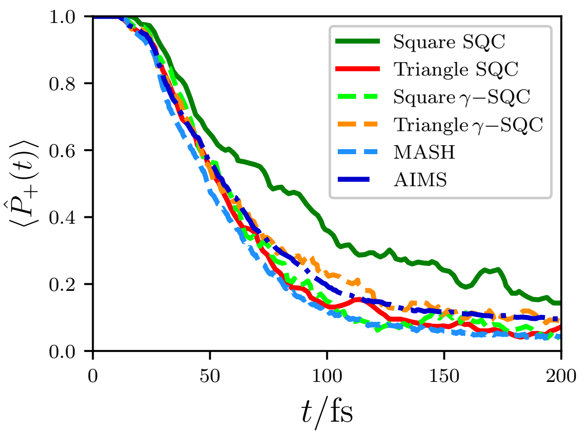

Figure S4: Figure showing the dynamical population of the upper adiabatic state in ethylene. The SQC results were taken from Ref. S17.

Nuclear Momenta in the Adiabatic Basis

In this section, we define the kinematic and canonical nuclear momenta in the adiabatic basis for quantum wavepacket approaches. An equivalent definition more applicable to semiclassical trajectory approaches can be found in Ref. S18.

In order to keep the resulting equations as simple as possible, we start with the general form of the Hamiltonian for an electron-nuclear coupled system expressed in a diabatic electronic basis

$$\hat{H} = \sum_j \frac{\hat{p}_j^2}{2m_j} + \hat{V}(\hat{\boldsymbol{q}}), \tag{S17a}$$

$$\hat{V}(\hat{\boldsymbol{q}}) = \sum_{kl} V_{kl}(\hat{\boldsymbol{q}}) \left| k \right\rangle \left\langle l \right| . \tag{S17b}$$

Here, $\hat{p}_j$ is the momentum operator for nuclear degree of freedom j of mass m_j and $\hat{V}(\hat{\boldsymbol{q}})$ is the potential matrix evaluated in a diabatic basis, $|k\rangle$, which is independent of the nuclear coordinates. From this expression for the Hamiltonian, the equation of motion for the expectation value of the nuclear momentum operator can be obtained as follows

$$\begin{aligned} \frac{\mathrm{d}}{\mathrm{d}t} \langle \hat{p}_j \rangle &= \frac{i}{\hbar} \langle [\hat{H}, \hat{p}_j] \rangle \\ &= - \left\langle \hat{V}'(\hat{\boldsymbol{q}}) \right\rangle , \end{aligned} \tag{S18}$$

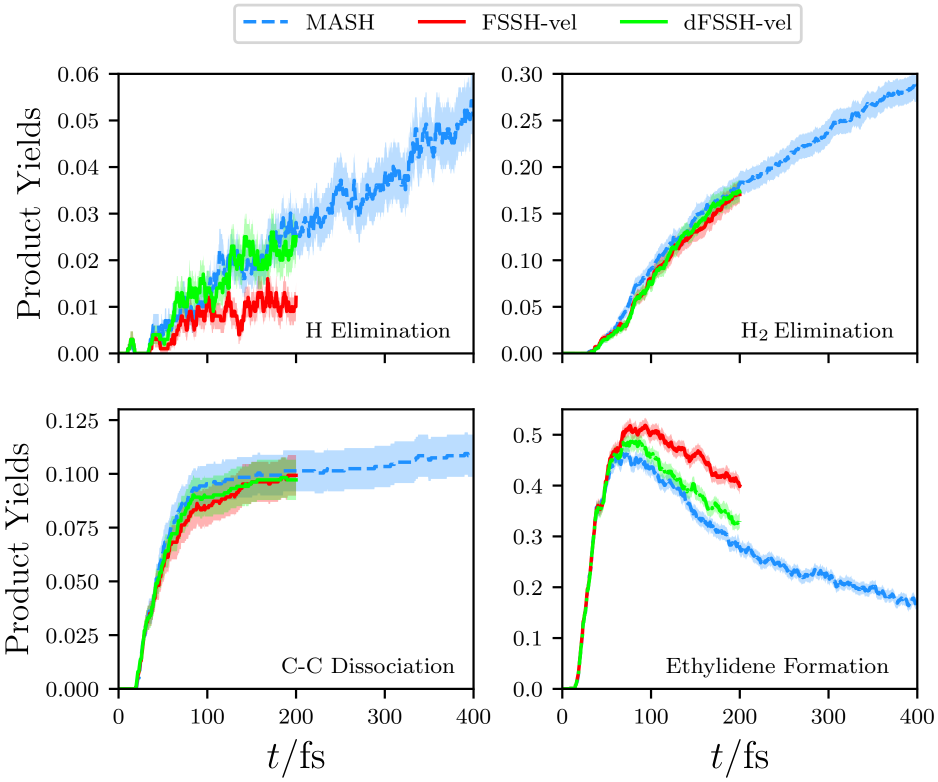

Figure S5: Dynamical product yields for the major products in the photodissociation of ethylene, calculated using FSSH-vel and dFSSH-vel. The MASH result is the same as in Fig. 4 of the main paper. The width of the shading represents twice the standard error.

where

$$\hat{V}'(\hat{\boldsymbol{q}}) = \sum_{kl} V'_{kl}(\hat{\boldsymbol{q}}) \left|k\right\rangle \left\langle l\right| , \tag{S19}$$

and $V'_{kl}(\boldsymbol{q}) = \frac{\partial V_{kl}(\boldsymbol{q})}{\partial q_j}$.

In order to obtain an analogous expression for the adiabatic basis, we express the full quantum state of the coupled electron-nuclear system using the Born-Huang expansion

$$\left|\psi(t)\right\rangle = \sum_{\lambda} \int \mathrm{d}\boldsymbol{q}\, \chi^{(\lambda)}(\boldsymbol{q}, t) \left|\psi_\lambda(\boldsymbol{q})\right\rangle \left|\boldsymbol{q}\right\rangle , \tag{S20}$$

where $\left|\psi_\lambda(\boldsymbol{q})\right\rangle$ is the electronic state associated with adiabat λ at nuclear coordinate $\boldsymbol{q}$, $\left|\boldsymbol{q}\right\rangle$ is the eigenstate of the nuclear position operator and $\chi^{(\lambda)}(\boldsymbol{q}, t)$ is the nuclear wavefunction associated with adiabat λ. For example in AIMS, $\chi^{(\lambda)}(\boldsymbol{q}, t)$ takes the form of a linear combination of Gaussian functions.

In order to evaluate the right-hand side of Eq. (S18) in the adiabatic basis, for which $\hat{V}(\boldsymbol{q})\,|\psi_\lambda(\boldsymbol{q})\rangle = V_\lambda(\boldsymbol{q})\,|\psi_\lambda(\boldsymbol{q})\rangle$, we can use the fact that

$$\langle\psi_\mu(\boldsymbol{q})|\hat{V}'(\boldsymbol{q})|\psi_\lambda(\boldsymbol{q})\rangle = \frac{\partial V_\lambda(\boldsymbol{q})}{\partial q_j}\delta_{\mu\lambda} - V_\lambda(\boldsymbol{q})\left\langle \frac{\partial\psi_\mu(\boldsymbol{q})}{\partial q_j}\middle|\psi_\lambda(\boldsymbol{q})\right\rangle - V_\mu(\boldsymbol{q})\left\langle\psi_\mu(\boldsymbol{q})\middle|\frac{\partial\psi_\lambda(\boldsymbol{q})}{\partial q_j}\right\rangle. \quad \text{(S21)}$$

Additionally using $d_j^{(\mu,\lambda)}(\boldsymbol{q}) = \left\langle\psi_\mu(\boldsymbol{q})\middle|\frac{\partial\psi_\lambda(\boldsymbol{q})}{\partial q_j}\right\rangle = -\left\langle\frac{\partial\psi_\mu(\boldsymbol{q})}{\partial q_j}\middle|\psi_\lambda(\boldsymbol{q})\right\rangle$ leads to the expression given by Eq. (1) in the main paper.

Finally, the left-hand side of Eq. S18 can be evaluated in the adiabatic basis to give

$$\langle\hat{p}_j\rangle = \sum_\lambda \langle\chi^{(\lambda)}(t)|\hat{p}_j|\chi^{(\lambda)}(t)\rangle + 2\hbar\sum_\lambda\sum_{\mu>\lambda}\mathrm{Im}\left[\langle\chi^{(\mu)}(t)|d_j^{(\mu,\lambda)}(\hat{\boldsymbol{q}})|\chi^{(\lambda)}(t)\rangle\right] \quad \text{(S22)}$$

where $|\chi^{(\lambda)}(t)\rangle = \int \mathrm{d}\boldsymbol{q}\,\chi^{(\lambda)}(\boldsymbol{q},t)\,|\boldsymbol{q}\rangle$. The first term on the right-hand side of this expression corresponds to the nuclear momenta associated with the nuclear wavefunctions on each adiabat, which is referred to as the conjugate momenta in the adiabatic basis. The total momenta, which also contains a contribution arising from the nuclear coordinate dependence of the adiabatic states, is referred to as the kinematic momenta. Defining $\langle\hat{p}_j^{\mathrm{ad}}\rangle = \sum_\lambda\langle\chi^{(\lambda)}(t)|\hat{p}_j|\chi^{(\lambda)}(t)\rangle$, Eq. (S22) can also be expressed in operator form as

$$\hat{p}_j = \hat{p}_j^{\mathrm{ad}} + \hbar\sum_\lambda\sum_{\mu>\lambda} d_j^{(\mu,\lambda)}(\hat{\boldsymbol{q}})\hat{\sigma}_y^{(\mu,\lambda)}(\hat{\boldsymbol{q}}), \quad \text{(S23)}$$

where $\hat{\sigma}_y^{(\mu,\lambda)}(\hat{\boldsymbol{q}}) = -i(|\psi_\mu(\hat{\boldsymbol{q}})\rangle\langle\psi_\lambda(\hat{\boldsymbol{q}})| - |\psi_\lambda(\hat{\boldsymbol{q}})\rangle\langle\psi_\mu(\hat{\boldsymbol{q}})|)$. This means that we could have equivalently derived Eq. (1) in the main paper using the adiabatic basis from the start as follows

$$\frac{\mathrm{d}}{\mathrm{d}t}\left\langle\hat{p}_j^{\mathrm{ad}} + \hbar\sum_\lambda\sum_{\mu>\lambda}d_j^{(\mu,\lambda)}(\hat{\boldsymbol{q}})\hat{\sigma}_y^{(\mu,\lambda)}(\hat{\boldsymbol{q}})\right\rangle = \frac{i}{\hbar}\left\langle\left[\hat{H}^{\mathrm{ad}}, \hat{p}_j^{\mathrm{ad}} + \hbar\sum_\lambda\sum_{\mu>\lambda}d_j^{(\mu,\lambda)}(\hat{\boldsymbol{q}})\hat{\sigma}_y^{(\mu,\lambda)}(\hat{\boldsymbol{q}})\right]\right\rangle, \quad \text{(S24a)}$$

$$\hat{H}^{\mathrm{ad}} = \sum_j\frac{\left(\hat{p}_j^{\mathrm{ad}} + \hbar\sum_\lambda\sum_{\mu>\lambda}d_j^{(\mu,\lambda)}(\hat{\boldsymbol{q}})\hat{\sigma}_y^{(\mu,\lambda)}(\hat{\boldsymbol{q}})\right)^2}{2m_j} + \sum_\lambda V_\lambda(\hat{\boldsymbol{q}})\hat{P}_\lambda(\hat{\boldsymbol{q}}). \quad \text{(S24b)}$$

References

(S1) Werner, H.-J.; Knowles, P. J.; Knizia, G.; Manby, F. R.; Schütz, M., *et al.* MOLPRO, version 2012.1, a package of ab initio programs. 2012; `http://www.molpro.net`.

(S2) Gómez, S.; Spinlove, E.; Worth, G. Benchmarking non-adiabatic quantum dynamics using the molecular Tully models. *Phys. Chem. Chem. Phys.* **2024**, *26*, 1829–1844.

(S3) Frisch, M. J.; Trucks, G. W.; Schlegel, H. B.; Scuseria, G. E.; Robb, M. A.; Cheeseman, J. R.; Scalmani, G.; Barone, V.; Petersson, G. A.; Nakatsuji, H.; Li, X.; Caricato, M.; Marenich, A. V.; Bloino, J.; Janesko, B. G.; Gomperts, R.; Mennucci, B.; Hratchian, H. P.; Ortiz, J. V.; Izmaylov, A. F.; Sonnenberg, J. L.; Williams-Young, D.; Ding, F.; Lipparini, F.; Egidi, F.; Goings, J.; Peng, B.; Petrone, A.; Henderson, T.; Ranasinghe, D.; Zakrzewski, V. G.; Gao, J.; Rega, N.; Zheng, G.; Liang, W.; Hada, M.; Ehara, M.; Toyota, K.; Fukuda, R.; Hasegawa, J.; Ishida, M.; Nakajima, T.; Honda, Y.; Kitao, O.; Nakai, H.; Vreven, T.; Throssell, K.; Montgomery, J. A., Jr.; Peralta, J. E.; Ogliaro, F.; Bearpark, M. J.; Heyd, J. J.; Brothers, E. N.; Kudin, K. N.; Staroverov, V. N.; Keith, T. A.; Kobayashi, R.; Normand, J.; Raghavachari, K.; Rendell, A. P.; Burant, J. C.; Iyengar, S. S.; Tomasi, J.; Cossi, M.; Millam, J. M.; Klene, M.; Adamo, C.; Cammi, R.; Ochterski, J. W.; Martin, R. L.; Morokuma, K.; Farkas, O.; Foresman, J. B.; Fox, D. J. Gaussian 16 Revision C.01. 2016; Gaussian Inc. Wallingford CT.

(S4) Plasser, F.; Ruckenbauer, M.; Mai, S.; Oppel, M.; Marquetand, P.; González, L. Efficient and Flexible Computation of Many-Electron Wave Function Overlaps. *J. Chem. Theory Comput.* **2016**, *12*, 1207–1219.

(S5) Mai, S.; Richter, M.; Heindl, M.; Menger, M. F. S. J.; Atkins, A.; Ruckenbauer, M.; Plasser, F.; Oppel, M.; Marquetand, P.; Gonzáalez, L. SHARC2.0: Surface Hopping Including Arbitrary Couplings — Program Package for Non-Adiabatic Dynamics. https://sharc-md.org/, 2018.

(S6) Mai, S.; Marquetand, P.; González, L. Nonadiabatic dynamics: The SHARC approach. *WIREs Comput. Mol. Sci.* **2018**, *8*, e1370.

(S7) Levine, B. G.; Coe, J. D.; Virshup, A. M.; Martínez, T. J. Implementation of ab initio multiple spawning in the Molpro quantum chemistry package. *Chem. Phys.* **2008**, *347*, 3–16.

(S8) Curchod, B. F. E.; Sisto, A.; Martínez, T. J. Ab Initio Multiple Spawning Photochemical Dynamics of DMABN Using GPUs. *J. Phys. Chem. A* **2017**, *121*, 265–276.

(S9) Ibele, L. M.; Curchod, B. F. E. Dynamics near a conical intersection—A diabolical compromise for the approximations of ab initio multiple spawning. *J. Chem. Phys.* **2021**, *155*, 174119.

(S10) Subotnik, J. E.; Jain, A.; Landry, B.; Petit, A.; Ouyang, W.; Bellonzi, N. Understanding the surface hopping view of electronic transitions and decoherence. *Annu. Rev. Phys. Chem.* **2016**, *67*, 387–417.

(S11) Granucci, G.; Persico, M. Critical appraisal of the fewest switches algorithm for surface hopping. *J. Chem. Phys.* **2007**, *126*, 134114.

(S12) Granucci, G.; Persico, M.; Zoccante, A. Including quantum decoherence in surface hopping. *J. Chem. Phys.* **2010**, *133*, 134111.

(S13) Mannouch, J. R.; Richardson, J. O. A mapping approach to surface hopping. *J. Chem. Phys.* **2023**, *158*, 104111.

(S14) Mannouch, J. R.; Richardson, J. O. A mapping approach to surface hopping. *J. Chem. Phys.* **2023**, *158*, 104111.

(S15) Ibele, L. M.; Curchod, B. F. E. A molecular perspective on Tully models for nonadiabatic dynamics. *Phys. Chem. Chem. Phys.* **2020**, *22*, 15183–15196.

(S16) Barbatti, M.; Ruckenbauer, M.; Lischka, H. The photodynamics of ethylene: A surface-hopping study on structural aspects. *J. Chem. Phys.* **2005**, *122*, 174307.

(S17) Weight, B. M.; Mandal, A.; Huo, P. Ab initio symmetric quasi-classical approach to investigate molecular Tully models. *J. Chem. Phys.* **2021**, *155*, 084106.

(S18) Cotton, S. J.; Liang, R.; Miller, W. H. On the adiabatic representation of Meyer-Miller electronic-nuclear dynamics. *J. Chem. Phys.* **2017**, *147*, 064112.
